# Attitudinal and Behavioral Characteristics Predict High Risk Sexual Activity in Rural Tanzanian Youth

**DOI:** 10.1371/journal.pone.0099987

**Published:** 2014-06-13

**Authors:** Stephen R. Aichele, Monique Borgerhoff Mulder, Susan James, Kevin Grimm

**Affiliations:** 1 Department of Psychology, University of California Davis, Davis, California, United States of America; 2 Department of Psychology, University of Geneva, Geneva, Switzerland; 3 Department of Anthropology, University of California Davis, Davis, California, United States of America; 4 Savannas Forever, Arusha, Tanzania; 5 Wissenschaftskolleg zu Berlin, Berlin, Germany; 6 The Whole Village Project, University of Minnesota, Minneapolis, Minnesota, United States of America; Alberta Provincial Laboratory for Public Health/University of Alberta, Canada

## Abstract

The incidence of HIV infection in rural African youth remains high despite widespread knowledge of the disease within the region and increasing funds allocated to programs aimed at its prevention and treatment. This suggests that program efficacy requires a more nuanced understanding of the profiles of the most at-risk individuals. To evaluate the explanatory power of novel psychographic variables in relation to high-risk sexual behaviors, we conducted a survey to assess the effects of psychographic factors, both behavioral and attitudinal, controlling for standard predictors in 546 youth (12–26 years of age) across 8 villages in northern Tanzania. Indicators of high-risk sexual behavior included HIV testing, sexual history (i.e., virgin/non-virgin), age of first sexual activity, condom use, and number of lifetime sexual partners. Predictors in the statistical models included standard demographic variables, patterns of media consumption, HIV awareness, and six new psychographic features identified via factor analyses: personal vanity, family-building values, ambition for higher education, town recreation, perceived parental strictness, and spending preferences. In a series of hierarchical regression analyses, we find that models including psychographic factors contribute significant additional explanatory information when compared to models including only demographic and other conventional predictors. We propose that the psychographic approach used here, in so far as it identifies individual characteristics, aspirations, aspects of personal life style and spending preferences, can be used to target appropriate communities of youth within villages for leading and receiving outreach, and to build communities of like-minded youth who support new patterns of sexual behavior.

## Introduction

Over five million youth between the ages of 15–24 are currently estimated to be living with HIV/AIDS worldwide, with new infections in this group occurring at a rate of 6000 per day. Sub-Saharan Africa is home to the majority of these youth, and Tanzania has been one of the countries most affected by the disease [1]–[3]. The earliest known HIV infections in Tanzania occurred in 1983 [4], and by 1998 the Tanzania National AIDS Control Program estimated that 1.63 million people (approximately 10%) over the age of 15 were infected with HIV [5]–[6]. National surveys conducted between 2003 and 2007/2008 indicated a moderate decline in HIV prevalence, from 7.0 to 5.7 percent, among 15- to 49-year-olds [4], [7], and HIV incidence also declined, at least for men [8]. This decline can be attributed to behavioral changes in the general population rather than to deaths of infected individuals [4]. Treatment and prevention programs launched during the 1990s and early 2000s likely played an important role in shaping these behavioral changes by providing increased access to condoms, counselling, and health education about HIV/AIDS prevention [3], [9].

Unfortunately, not all sectors of the population benefitted from these interventions: Infections continue to be a serious problem, concentrating primarily in rural areas among youth and those with limited education [8], [9], as is generally the case in sub Saharan Africa [10], [11]. In 2012, HIV prevalence estimates for the general population of Tanzania were 3.4% for females and 1.4% for males [4], with elevated estimates for persons aged 20–24 (4.4% for females, and 1.7% for males) [3], [12]. This situation likely reflects a combination of the extended adolescence afforded by primary education (that typically lasts until 15–16 years of age in rural villages), the transactional nature of sexual relations, conflicting traditional and contemporary norms, poverty, peer pressure, ambition, and opportunism [13].

Sexual risk-taking behaviors are known to be the primary drivers of the HIV epidemic. Although the proportion of 15–19-year-old Tanzanians reporting two or more partners declined between 2004 and 2008 [7], condom use is still low among this age group, with less than one third of young people reporting having used a condom during their first sexual experience. And the incidence of unprotected sex among Tanzania’s youth is particularly high in comparison to that of youth in other countries [14]. Furthermore, young people infected with HIV are known to exhibit a higher degree of sexual risk-taking behavior than the general population - both prior to and following diagnosis [3], [15]–[18]. Sadly, the very psychosocial and socio-economic influences that place young people at risk for acquiring HIV also inhibit their ability to access treatment and prevention resources [19].

School-based sex educational programs have become a popular strategy in HIV-related public health campaigns – for Tanzania, see for example [20]. These are nevertheless hindered by the typically authoritarian teacher-student relationship, low attendance, poor literacy skills, and not infrequent sexual abuse in school [13]. Comparative evaluations of such initiatives show that such interventions produce very mixed outcomes [10], [21], often failing to live up to expectations for behavioral change, even when knowledge and attitudes have been positively transformed [22]. Even more generally, strategies focused on youth seem to be having only limited success: In a recent meta-analysis, Michielsen et al. [23] reviewed the efficacy of HIV-prevention interventions in changing sexual behavior of young people in sub-Saharan Africa and concluded that current HIV education strategies had few of the desired positive impacts on behavior, with the exception of increased condom use among males. There was otherwise no evidence that these educational interventions significantly reduce risk-taking behavior, despite significant changes in HIV-related knowledge and attitudes.

Recent ethnographic research on the sexual behavior in rural villages of Tanzania’s Mwanza Region paints a rich qualitative picture of how youth negotiate sexual partnership to secure personal, economic and hedonistic goals, even after marriage. These sexual relations are influenced by age, social status, peer pressure, ambition and opportunism, as well as by poverty [13]. Such studies, and the research reviewed therein, highlight how the continuing AIDS epidemic depends on both structural factors (poor schooling, limited health services, few recreational options for youth, women’s restricted economic options) and cultural factors (a lack of concern regarding diseases with long time frames, alcoholism, fatalism, opportunism, and “double standards” for men and women), and in particular the conflict that youth face in reconciling traditional values with more contemporary demands as they negotiate their educationally-extended adolescence.

**Figure 1:**
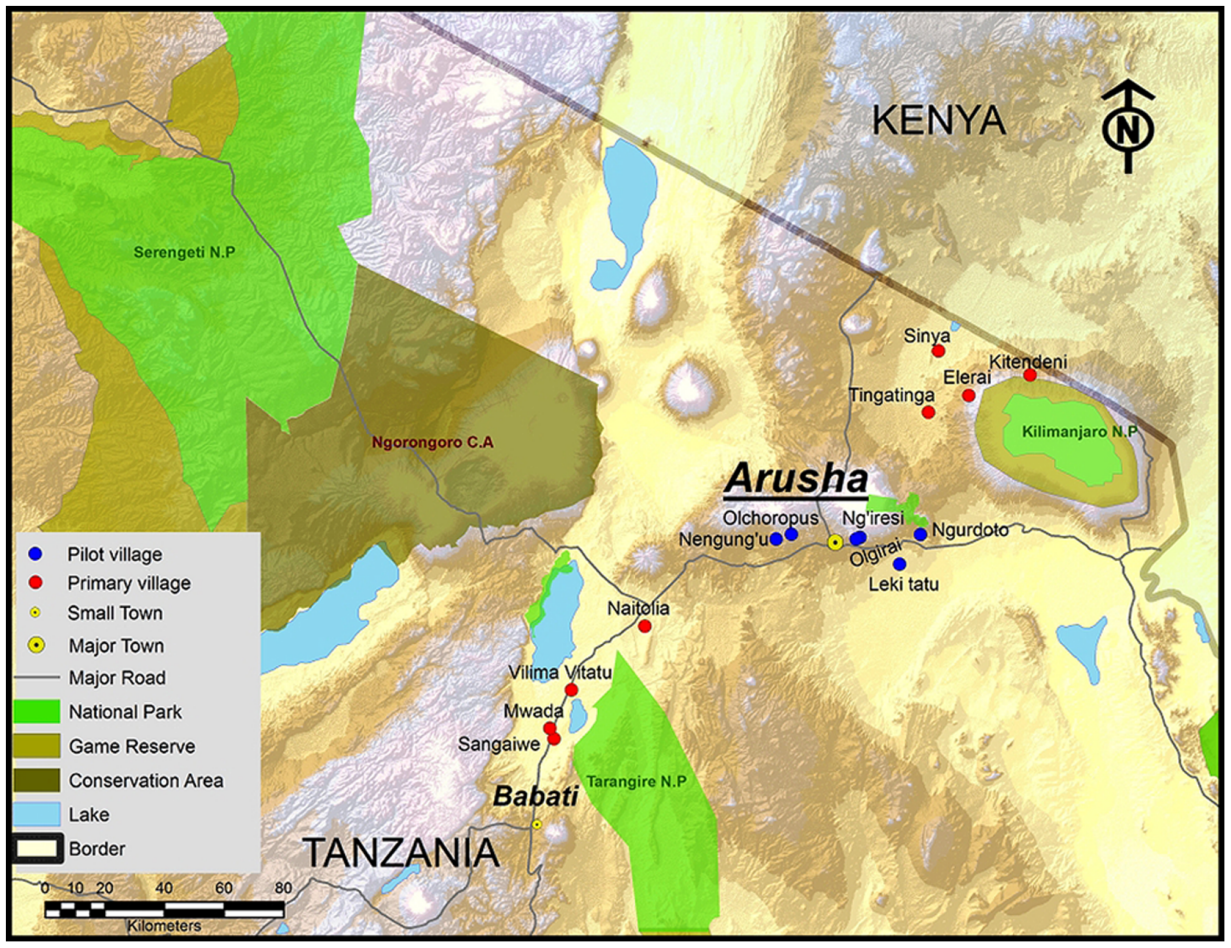
Map Showing the Villages in which the Youth Survey was Conducted. Red filled circles indicate primary study villages (#s 7–14), and blue filled circles indicate pilot study villages (#s 1–6).

Given the conjunction of continued high HIV transmission rates among youth, poorly performing educational/awareness interventions, and the complex structural and cultural factors that continue to expose youth to such high rates of infection despite widespread knowledge of the disease, we decided to broaden the investigation of factors influencing sexual behavior of youth to include not only HIV knowledge but a range of attitudinal and behavioral measures, collectively termed psychographic features, that are likely to affect an individual’s risk of HIV infection. We administered our survey to 546 youth across 8 Tanzanian villages (see Figure 1). The psychographic characteristics (identified through dimension reduction during a pilot survey) consisted of personal vanity, family building values, ambition for higher education, town recreation, perceived parental strictness, and preferences for spending on the family. We examined these factors as predictive of sexual risk-taking behavior within a set of models that included basic demographic measures (age, sex, education and employment), village membership, and media exposure. Our selection of these latter variables was informed by current understanding of the patterning of the prevalence of HIV in sub Saharan Africa, and particularly in Tanzania (see Section E in File S1for additional information pertaining to demographic predictors of HIV prevalence). Additionally, we included HIV awareness in the models as a predictor variable in light of the current concern that school-based educational interventions are not effective in changing behavior.

## Methods

### Survey Development and Administration

Psychographic research has not previously been used in Tanzania. To develop our research methodology and survey, we reviewed literature of the Tanzanian HIV-AIDS epidemic, consulted with focus groups, interviewed experts, and engaged in informal discussion with local youth. Focus groups consisted of Peace Corps volunteers teaching in schools (primary and secondary) and Tanzanian college graduates ranging in age from 24 to 30. Expert interviews were held with district education officers and village teachers. Informal discussions occurred among the project team and youth in such places as secondary schools, pool halls, and village centers. We conducted pilot and primary surveys (results from the latter are reported here) in predominantly Maasai areas of northern Tanzania (54% of those sampled).

Consent was requested of subjects, and when minors also from next of kin. Consent was oral rather than written because this is most appropriate in rural Tanzanian communities with limited literacy skills, and where many individuals harbor mistrust of written documentation. Participant consent was recorded in separate documentation. Age, ethnicity and gender were recorded through culturally-appropriate means, and participant anonymity was maintained during data analysis (i.e., via replacement of personal identifiers with ID numbers prior to analysis). This research methodology was approved by the Human Research Protection Program, University of Minnesota "The Whole Village Project"/IRB Code Number: 0905S65241” and by the National Medical Research Institute in Dar es Salaam.

Outcomes from the pilot study (described in Section A of File S1) informed further refinements of the survey’s content and its administration (i.e., the interview process). For example, the gender bias in response patterns to several items in the pilot survey (particularly % non-virgins: See Table A in File S1) mandated our subsequent use of nonverbal response cards [24] during the interview process in order to mask participants’ responses to sensitive items (for further details, see Discussion). We administered the revised questionnaire (Supplemental Digital Content 2) to 546 youth participants located in eight rural villages in Arusha, Longido, Babati and Monduli Districts in March 2011. Demographic data obtained from these individuals are provided in Table 1.

**Table 1:** Participant Demographics.

Statistic	Value	
N	546	
Number of Villages	8	
% Female	48	
Mean Age (min, max)	16.8 (12, 25)	
Highest Education Completed		
% None	18	
% Primary School	58	
% Secondary School	24	
Employment Status		
% Unemployed	29	
% Farming	64	
% Off-Farm Work a	7	
Ethnicity		
% Maasai	54	
% Mbugwe	21	
% Arusha	8	
% Other	17	
	female	Male
% Tested for HIV b	31.7	23.9
% Non-Virgins	44.7	40.8
% Used a Condom During Last Sex c	7.6	12.0
Mean (SD) Age at First Sex c	14.6 (3.8)	14.2 (4.2)
Mean (SD) # Lifetime Sexual Partners c	2.0 (1.7)	3.1 (4.1)

a Almost invariably this is in addition to farming.

b Non-responders were included in calculation of percentage as 'untested'.

c Only those who reported being sexually active were included in the calculation.

### Dimensionality Reduction of Psychographic Data

The survey included 71 ordinal items (grouped into 6 categories) to assess psychographic features of the participants (e.g., such as personal values and motives), 20 dichotomous items related to spending preferences, and 10 dichotomous items for the purpose of assessing HIV awareness. We employed a two-step factor analytic approach [25] to reduce the dimensionality of the data related to these 101 observed variables down to 7 factors: We first examined the pilot data via exploratory methods, and we then applied structural (i.e., “confirmatory”) methods to the psychographic data from the primary survey. This resulted in the following factors: (a) HIV awareness (indicated by responses to three most informative questions related to HIV prevention and transmission: condom use, monogamy, and food sharing); (b) "Personal Vanity" (indicated by the valuation of power, bravery, and sex appeal as personal attributes); (c) "Family-Building Values" (indicated by desires for marriage, building a house, and children); (d) "Ambition for Higher Education" (indicated by survey items related to desired scholastic achievement); (e) “Town Recreation" (indicated by preferences for dancing/nightclubbing, pool playing, and drinking alcohol as leisure preferences – note, these activities reflect social preferences pursuable in villages but adopted from town); and (f) "Perceived Parental Strictness" (indicated by items asking respondents how they thought their parents would react upon learning the respondent had a boy/girlfriend, had spent the night out, or were pregnant/had gotten someone pregnant); and (g) "Preferred Spending on Family" as indicated by the stated willingness to spend on food for family members given low, moderate, and high levels of available funds. We collectively refer to factors 2 through 7 as "psychographic" characteristics. For additional information related to these analyses, see Sections A and B and Tables B-G in File S1).

### Psychographic Factors as Predictive of HIV/AIDS-related Outcomes

Our central research objective was to determine whether HIV awareness and psychographic factors provided information beyond that obtained from basic demographic variables (e.g., age, gender, education, ethnicity, media access) in predicting the five variables related to sexual behavior and HIV risk (see Table 2). For each of the 5 outcome variables, we conducted a series of hierarchical regression analyses in which we first fit a baseline (intercept only) model (M0) to the data. We then sequentially added the following sets of predictors and assessed change in model fit: (M1) basic demographic variables (age, gender, highest level of completed education, employment status, Maasai/non-Maasai ethnicity), (M2) village membership (8 villages), (M3) average weekly media consumption (television, radio, print media), (M4) HIV knowledge, (M5) psychographic features (personal vanity, family values, educational ambition, town leisure, perceived parental strictness, and concern for family as indicated by the stated willingness to spend on food for family members). See SDC1.3, for methodological details of this analysis.

**Table 2:** Hierarchical Regression Analyses.

		Model Fit a by Sets of Predictors					
	N	M0	M1	M2	M3	M4	M5
		*null model*	*demographics*	*village*	*media use*	*HIV knowledge*	*psychographics*
			D *df* = 7	D *df* = 7	D *df* = 3	D *df* = 1	D *df* = 6
*Outcome Behavior*		*Logistic Regression*					
Tested for HIV?	538	634.9	483.4*	479.8	479.6	470.1*	451.2 [.46] *
Ever sexual?	536	732.2	559.8*	556.7	553.8	558.4	531.3 [.42] *
Condom used last sex? b	226	248.5	206.1*	191.9*	185.0	186.3[.45] *	181.0
		*Poisson Regression*					
Life sexual partners? b	230	1105.6	1042.2*	997.6*	991.5	968.4*	945.5*
		*Survival Analysis*					
Age of virginity loss? b ^,^ c	219	1228.0	1187.5*	1178.4	1183.7	1186.2	1185.4

*Note*. Sets of predictors were added in sequence from M0 to M7. M0  =  Null Model (intercept only), M1 =  demographic variables (age, gender, highest education achieved, employment status, Maasai vs. other ethnicity), M2 =  village membership, M3 =  weekly media consumption (radio, television, print media), M4 =  HIV knowledge, M5 =  psychographic factors. Change in model fit was assessed via likelihood ratio testing: i.e., change in deviance relative to change in degrees of freedom. Sets shown to improve model fit (* p<.05) were carried forward in subsequent analyses.

a Deviance (-2*log-likelihood) is reported for each model. R^2^ is reported in brackets for best-fitting logistic models.

b Analyses were carried out on data from the subset of individuals who reported previous sexual activity.

c Age was not included in the set of basic demographic variables in this analysis.

## Results

In all analyses, addition of demographic variables (M1) improved fit beyond that of the baseline model (Table 2). Village membership (M2) proved useful in predicting condom use and number of lifetime sexual partners. Inclusion of weekly media consumption as a predictor (M3) did not improve model fit for any of the outcome variables. Inclusion of HIV knowledge (M4) improved prediction of the following outcomes: HIV testing, condom use, and number of lifetime sexual partners. Psychographic characteristics (M5) improved model fit for the outcomes of HIV testing, non-virginity, and number of lifetime sexual partners. We report parameter estimates (raw regression weights and standard errors) from the best-fitting models for each outcome variable in Table 3, both for psychographic characteristics and for the more conventional predictors.

**Table 3:** Predictors of Sexual-Risk Taking.

Grouped Predictors	*Tested for HIV?* a	*Ever Sexual?* a	*Condom Used Last Sex* a ^,^ b	*# Lifetime Sex Partners* b ^,^ c	*Virginity Loss 'Hazard'* b ^,^ d
*Intercept*	−8.38 (.91) *	−5.76 (.70) *	−3.54 (1.91)	−1.73 (.42) *	–
*Psychographic Factors*					
Personal Vanity	−.18 (.18)	.27 (.17)	–	−.03 (.08)	–
Ambition for Higher Education	−.05 (.21)	−.19 (.19)	–	−.23(.08) *	–
Family-Building Values	.83 (.30) *	.54 (.27) *	–	−.03 (.12)	–
Town Recreation	−.22 (.33)	.52 (.31)	–	.42 (.09) *	–
Perceived Parental Strictness	.23 (.22)	.11 (.21)	–	−.10 (.08)	–
Prefer Spending on Family	.56(.24) *	.52 (.24) *	–	−.06 (.09)	–
*Demographics*					
Age	.29 (.04) *	.30 (.04) *	.02 (.07)	.05 (.01) *	e
Gender: Female	1.12 (.31) *	.77 (.28) *	−.64 (.55)	−.59 (.13) *	−.16 (.15)
Education					
Primary	1.09 (.40) *	−.54 (.31)	.77 (.69)	.34 (.13) *	−.57 (.19) *
Secondary	1.33 (.47) *	−1.12 (.39) *	2.01 (.83) *	.34 (.18)	−.73 (.23) *
Employment					
Farming	.45 (.30)	.22 (.26)	1.65 (.88)	−.11 (.15)	−.25 (.18)
Off-farm	.67 (.49)	.24 (.48)	1.31 (1.02)	−.03 (.19)	−.74 (.28) *
Ethnicity: Maasai	.64 (.27) *	.56 (.24) *	−.53 (1.05)	1.5 (.24) *	.35 (.16) *
*Village Membership*	–	–	1^f^	3^f^	–
*Media Consumption* g	–	–	–	–	–
*HIV Knowledge*	.83 (.21) *	–	.72 (.36) *	−.39 (.07) *	–

*Note*. Raw regression weights are reported with standard errors (in parentheses). Dashes (−) reflect sets of items that were not included in an analysis due to their negligible contribution to improvement in model fit. * *p*<.05

a Regression weights represent change in log odds (e.g.,.77 gives *e^0^* ^.77^ = 2.16× increase in odds of engaging in sexual behavior for females relative to males, given other covariates in the model.

b Analyses carried out on data from the subset of individuals who reported previous sexual activity.

c Exponentiated coefficients show the multiplicative increase in expected number of lifetime sex partners (e.g.,.34 gives *e^0^* ^.34^ = 1.4× increase in number of sexual partners for youth with primary education relative to those without).

d Coefficients represent change in log odds of incremental probability of virginity loss. As examples, holding other variables constant, [A] completion of secondary education reduces the incremental (yearly by age) hazard of virginity loss by a factor of *e* ^−0.73^ = 0.48 or 52% (1–.48) relative to those who have not completed primary school, and [B] Maasai have an increased yearly hazard of virginity loss equal to *e* ^0.35^ = 1.42 or 42% relative to non-Maasai.

e Age was not included in the set of basic demographic predictor variables for this analysis.

f Number of villages (out of 7, excluding reference village) showing a significant positive relationship to outcome.

g Media consumption is included for consistency, despite having been excluded as a predictor set in each of the best-fitting models in Table 2.

**Figure 2:**
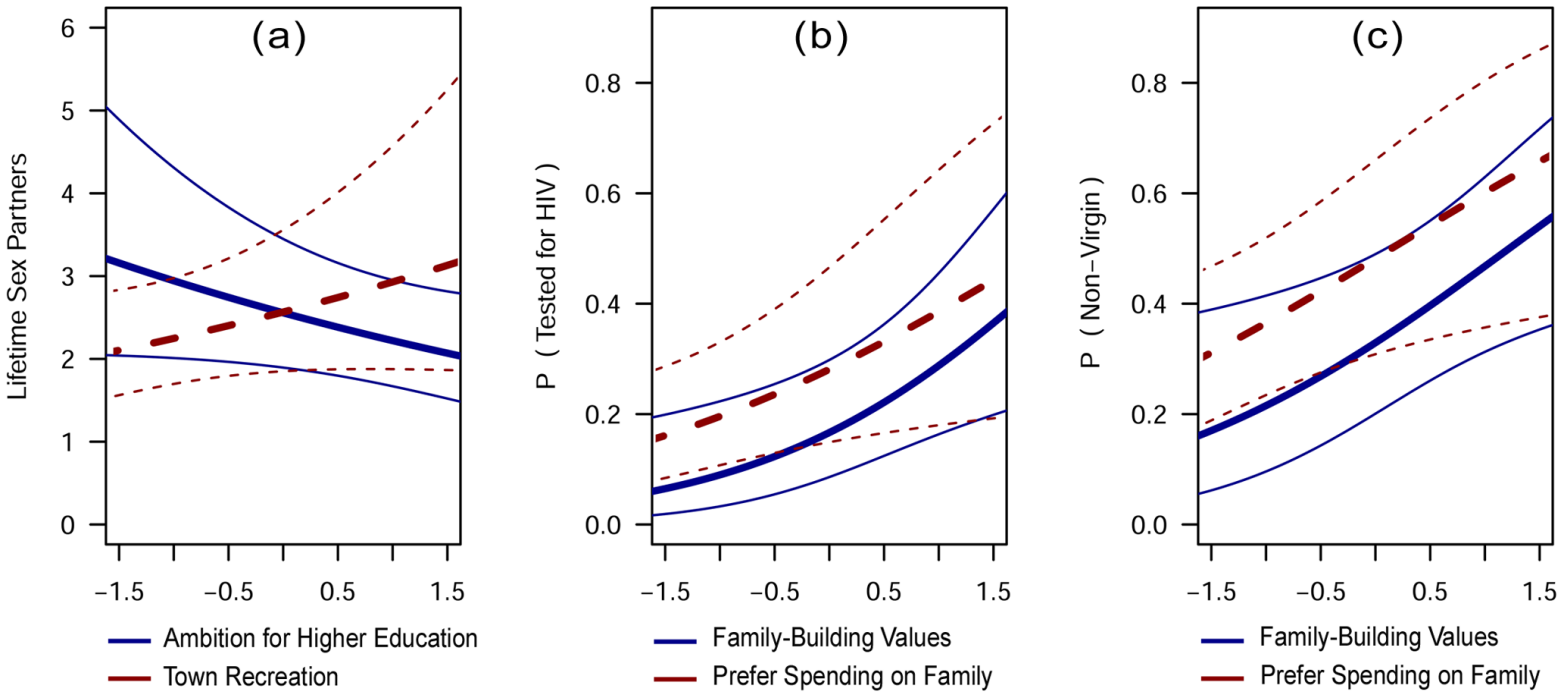
Selected Psychographic Characteristics and their Relationship with Sexual-Risk Outcomes. These partial regression plots [34] show the predicted influence of significant behavioral characteristics (x-axes) on sexual risk-taking outcomes (y-axes), controlling for demographic variables. Sexual risk-taking outcomes, by panel, include (a) number of lifetime sexual partners, (b) probability of previous HIV testing, and (c) probability of non-virgin status.

Several psychographic characteristics were significantly related to three of the outcome variables. Ambition for higher education was negatively linked to number of lifetime sexual partners. A preference for town recreation (dancing, playing pool, drinking alcohol) was positively related to number of lifetime sexual partners (Figure 2a). Family building values (i.e., desire for marriage, children, house building) and the preference to spend money on food for one's family were positively related both to prior HIV testing and to status as a non-virgin (Figures 2b and 2c, respectively). Personal vanity and perceived parental strictness were not significantly related to any of the outcome variables.

Turning now to the conventional variables, age was positively linked with having previously been tested for HIV, non-virginity (currently reported status), and number of lifetime sexual partners among sexually active individuals (Table 3). Females were more likely than males to report having been tested for HIV and to have previously engaged in sexual intercourse, but sexually active females reported having fewer sexual partners than sexually active males across their lifetimes. Compared to those with no education, youth who had completed primary or secondary education were more likely to have been tested for HIV and to have lost their virginity at an older age (as indicated by a relatively lower age-based hazard of virginity loss). Those who reported completion of secondary education were less likely to report having previously engaged in sex, and if they did report prior sexual activity, they were more likely to have used a condom during their last sexual encounter. Those who reported completing primary (but not secondary) education reported a greater number of lifetime sexual partners than those with no education. Off-farm employment was negatively predictive of the age-based incremental probability of virginity loss, indicating that youth working for a salary or wage lose their virginity at a later age. Maasai ethnicity was positively related to reported status as a non-virgin, age-based incremental probability of virginity loss, and number of lifetime sexual partners; however, Maasai individuals also reported higher levels of HIV testing. Village membership was a significant (positive) predictor of condom use in 1 village and of number of lifetime sexual partners in 3 villages. Media consumption was not associated with any outcome. Those who showed greater HIV knowledge were more likely to report having previously been tested for HIV and to have used a condom during their last sexual encounter. Among those who were sexually active, HIV knowledge was also negatively related to number of lifetime sexual partners.

## Discussion

Our principal novel finding (Table 2) is that psychographic characteristics of individuals are of value in statistically predicting three out of the five outcomes assessed: whether an individual tests for HIV, whether he or she becomes sexually active, and number of sexual partners – even after taking into account the effects of other more conventional predictors. This to our knowledge is the first time such variables have been investigated systematically in a multivariate hierarchical framework within this domain of research, and the results suggest that interventions designed to target these psychographic features would be valuable in rural areas where the pandemic is still growing. We first discuss these psychographic findings. We then examine the ancillary results showing that HIV knowledge has positive behavioral influences in this sample, an outcome that runs counter to conclusions of current reviews. Following this, we consider implications for intervention strategy. We relegate to Section E of File S1 discussion of the effects of conventional demographic and socioeconomic predictors (age, sex, education, employment, ethnicity, and village heterogeneity) insofar as these are largely in line with previous work, although the failure to show any effect of media consumption, and the relatively higher use of HIV testing by Maasai than by other ethnicities, are surprising outcomes (we attribute the latter, tentatively, to donor-particular interests in focusing on the Maasai population, but this needs further study).

Among results linked to psychographic characteristics, we first found that youth who are ambitious to achieve higher education have fewer sexual partners, controlling statistically for educational achievement. This is what one might expect among more ambitious and goal-orientated individuals who are presumably not heavily discounting the future and who would like to stay healthy, and this outcome is consistent with qualitative findings that youth who are educationally ambitious show aversion to risk taking [13]. While educated and wealthy people were initially most at risk from the HIV epidemic [9], our evidence of restricted sexual partners among youth with educational aspirations, even at the rural village level, is a welcome development. Furthermore, given evidence that the educated are more likely than the uneducated to get tested for HIV and less likely to be sexually active [9]; [26], this outcome confirms the widespread view that promoting education will help reduce the HIV epidemic.

Second, there are two psychographic traits that capture home-focused individuals – those who express a strong desire for marriage, children and building a house, and those who have a stated inclination to spend money on food for the family. These home-focused individuals are more likely to have tested for HIV status, and they are less likely to be virgins. This too is encouraging: Youth who are settling down, or who plan to settle down in conventional ways, are testing for HIV, and we find no evidence that these youth are losing their virginity at a particularly young age or showing a preference for a large number of sexual partners. The prognosis would be even better if individuals in this group had reported significantly fewer sex partners and/or showed more condom use than less home-focused individuals. Clearly HIV programs need to emphasize, for such home-focused individuals, the message that sticking with a single partner, and/or using condoms, would protect them further. This is important given that it is common for individuals in Tanzania to continue having multiple concurrent relations after their marriage [13] or to engage in rapid serial monogamy [27].

Finally, youth who like dancing, playing pool and drinking alcohol (those we termed as having a preference for town recreation), are more likely to have a large number of lifetime sexual partners compared to those who profess no such preferences. These youth are well known. They like visiting local towns (where they first experience these forms of recreation) and then come back to the village, with cool clothes and gadgets, to hang out at the village centers and popular drinking spots. They form cliques, and their behavior appears to be far more strongly influenced by their peers than by their kin [28]. They seem to have little sense of the risk to which they are exposing themselves which, as Macintyre et al. [29] show for South African youth, is typical among individuals who have weak ties to their families and their local communities. They use their “cool” to seduce others into sexual relations, such that they attain multiple partners, and they may act as patrons [30] to younger and more village-based youth who aspire to modernity and who are looking for relationships which will bring them a better life. From ethnographic observations, we often know these youth to be disillusioned with the promise of development and the better life they anticipated after completion of their education. Instead, when they return to their villages, where they find no employment opportunities and accordingly struggle with farming or opening small businesses (a kiosk, trading goods on the side of the road, bicycle repair, etc.), they become disheartened. Typically they resort to as much of a life of leisure as they can afford, attempting to lure others into their new habits.

Of ancillary interest are the findings that youth who are better informed about HIV are more likely to have been tested for HIV, to report having used a condom during sexual intercourse, and to have fewer sexual partners during their lifetimes. These results counter previous studies in which HIV/AIDS education has been shown to be a poor deterrent to high-risk sexual behaviors. Our results may differ from previous studies either because we control statistically for a range of significant demographic predictors or because the quality of HIV/AIDS education programs is particularly good in this part of Tanzania due to strong donor activity. Indeed, our village-level institutional analyses (unpublished data) show considerable HIV/AIDS outreach activity. This inter-village variation in HIV/AIDS outreach does not show a consistent association with village level HIV/AIDS knowledge (unpublished data), most likely because projects are targeted in communities where levels of ignorance about HIV/AIDS are suspected to be high, so baselines differ. Nevertheless, these findings, that youth who are better informed about HIV are less likely to engage in risky behaviors, provide a positive perspective on the effects of HIV/AIDS education. While causality is difficult to establish, and it is of course quite possible that those individuals who are sexually cautious (test for HIV, use condoms, and practice monogamy) pay more attention to and retain HIV/AIDS educational messages, it seems plausible to conclude that good HIV/AIDS outreach programs contribute to significant positive behavioral outcomes at the individual level. Longitudinal studies are needed to further corroborate this optimistic interpretation.

What light do these outcomes shed on potential intervention strategies? First, we have suggested that youth with high educational aspirations are behaving in ways that can curtail the spread of HIV/AIDS. These individuals (whom our ethnographic observations suggest are often found through “choir” or sports groups) could be selected for positions of youth leadership while still in secondary school. Health services outreach could support groups of such individuals in setting up clubs and by fostering social engagement through shared activities and visits among clubs of different villages. Second, although home-focused individuals (when compared to less home-focused individuals) are more likely to test for HIV, they report no fewer sexual partners nor greater condom use, suggesting the need to design specific messages for youth who are already settled, or contemplating settling, as couples. Such individuals can often be identified through the village school and health committees present in most Tanzanian villages. Finally there are the youth who like dancing, playing pool, and drinking alcohol. These young men and women are typically disengaged from trying to make a living in the village, and they are also known to exert considerable influence on their peers. A successful HIV/AIDS program for these individuals requires much more than just enhancing awareness and education: Vocational-training with tangible employment opportunities that give them direction and purpose will likely be of most benefit, with strong recruitment efforts made in the village “centers” where pool tables and bars are typically found.

While identifying individuals, or groups of individuals, at a village level might seem inordinately time consuming, what are the alternatives? Efforts to mitigate sexual risk-taking behaviors via "top-down" changes in socio-economic and institutional infrastructure, such as increased economic opportunities for women and improved educational and health facilities, require complex and expensive interventions that lie outside the remit of health educators who often work in local hospitals and NGOs with very limited resources. We therefore advocate, as a more tractable strategy, taking a "bottom up" approach toward reducing sexual risk-taking. This will require confronting maladaptive cultural, behavioral, and attitudinal norms (such as time discounting and alcohol consumption) and instilling ambition for higher education and resistance against harmful forms of peer pressure. Toward this end, the identification of individuals with strong educational ambition and/or a clear home-building focus is a necessary first step. Interventions can then aim at strengthening affiliations between these individuals so as to develop nodal points and networks from which beneficial behavioral and attitudinal changes may propagate to the broader culture. This approach of building communities of like-minded individuals to support one another in overcoming entrenched and deleterious cultural norms has proven effective in, for example, eradicating foot-binding in China [31] and in halting female genital modifications in parts of West Africa [32].

Before closing, we acknowledge the shortcomings of large scale survey work with respect to sensitive issues. Bias in self-reports stemming from secrecy, shame, and/or boastfulness is not uncommon in large-scale surveys, and self-report bias is of particular concern in studies of adolescents (e.g., [33]). This remains the case even when researchers utilize participant observation methods designed to reduce these sources of bias [13]. Although all of our interviewers were young, Tanzanian, and matched by sex with interviewees, we nevertheless observed sex-related biases in reporting during the pilot study. To address this problem, we subsequently utilized an innovative method developed by Lindstrom et al. [24] to obtain responses to those survey items found to be of concern. In this method, the interviewer is blind to the interviewees’ responses. Comparing the results of Table 1 (Primary study) with Table A of File S1 (Pilot study) we find that women are more than twice as likely to report non-virgin status when their response is concealed; They are also more likely to report having tested for HIV, a younger age at first sex (by 2.2 years), and a larger number of lifetime sexual partners. Men’s responses were almost identical across the surveys, with the exception of reporting fewer lifetime sexual partners when their response was concealed. While we appreciate that survey methods are not ideal for collecting data on sexual behavior, we believe that use of the Lindstrom et al. method is valuable for this purpose.

To summarize, we developed an inventory to assess demographic, dispositional and behavioral features of rural Tanzanian youth in order to better understand psychographic features linked to sexual risk-taking behaviors. We previously identified six psychographic features that we here examined as predictive of sexual risk-taking behavior. We found that four of these psychographic variables provided information beyond that obtained from more conventional demographic and socioeconomic measures alone. Specifically, individuals oriented toward higher education and/or home- and family-building reported a lower likelihood of engaging in sexual risk-taking behaviors, whereas individuals oriented toward hedonic pursuits (e.g., dancing, playing pool, consuming alcohol) were more likely to engage in sexual risk-taking and, presumably, therefore more likely to contract HIV/AIDS. At present, Tanzanian youth are subject to conflicting social norms that they derive from their distinct ethnic origins, their exposure to modernizing cultural trends, the all-too-often mixed messages they receive from school teachers, their economic constraints, and their idiosyncratic preferences [13]. Different individuals negotiate such conflicts in different ways, and these differences are likely reflected in their aspirations, views, and behaviors. We believe, and our results indicate, that these psychographic features hold valuable information about the determinants of sexual risk-taking behaviors linked to HIV transmission, and we hope that this knowledge proves beneficial in the design of future HIV/AIDS interventions.

## Supporting Information

File S1 **Additional analytic detail and discussion.** Section A Exploratory factor analyses of pilot survey data. Section B Structural factor analyses of primary survey data. Section C Hierarchical regression methodology. Section D Tables A-J. Section E. Discussion of age, sex, education, employment and ethnicity effects. Section F References.
(DOC)

File S2 **Survey administered to Tanzanian youth to assess demographic, attitudinal and behavioral characteristics in relation to HIV/AIDS outcomes.**
(DOC)
