# Supplementary material for: Attitudinal and Behavioral Characteristics Predict High Risk Sexual Activity in Rural Tanzanian Youth
Source: PLoS One. 2014 Jun 13;9(6):e99987. doi: 10.1371/journal.pone.0099987 (PMC4057388; doi:10.1371/journal.pone.0099987)
Supplement: File S1 — Additional analytic detail and discussion. Section A Exploratory factor analyses of pilot survey data. Section B Structural factor analyses of primary survey data. Section C Hierarchical regression methodology. Section D Tables A-J. Section E. Discussion of age, sex, education, employment and ethnicity effects. Section F References. (DOC) [file pone.0099987.s001.doc]

**S1: Additional analytic detail and discussion**

**List of Contents**

**Section A: Exploratory factor analyses of pilot survey data**

**Section B: Structural factor analyses of primary survey data**

**Section C: Hierarchical regression methodology**

**Section D: Tables**

**Section E: Discussion of age, sex, education, employment and ethnicity effects**

**Section F: References**

**Section A. Exploratory factor analyses of pilot survey data**

The pilot survey (conducted across 6 rural villages in Arusha and Meru Districts in November 2008) employed 190 items used to assess age, sex, education level, employment, home location, family configuration and support, primary language, travel patterns, media consumption, social and recreational activity preferences, perceived parental strictness, personal goals and aspirations, religious and other views, spending preferences, sexual behavior, valued personal characteristics (e.g., strength, beauty), drug use, and HIV-related knowledge. Pilot survey participants’ demographics information is provided in Table S1-1.

Due to the number of psychographic variables in our survey, we factor analyzed the ordinal items in two passes. In the first pass, we used parallel analysis [1] to determine the number of factors to extract within each category of items. These analyses were carried out using R statistical software [2] using the "Psych" package [3]. To approximate the number of latent dimensions of the pooled ordinal (non-dichotomous) items, we summed the resulting factor counts across categories (Table S1-2, "1^st^ pass") to arrive at a total of 15 possible factors. We then carried out an exploratory factor analysis of the 71 pooled ordinal items using Mplus statistical software [4] with unweighted least squares estimation for ordinal outcomes and Crawfer oblique rotation = 0.5. We chose to extract 10 factors rather than 15 given that our initial approximation of the maximum number of extractable factors did not account for possible item-factor loadings across categories. We then selected 3 or 4 items as indicators for each factor based on the strength of their factor loadings and thematic coherence. This resulted in a subset of 36 items. In the second pass, we applied parallel analysis to the pooled data for these 36 items to determine that up to 6 latent dimensions might reasonably account for the observed responses. We then factor analyzed this subset using robust weight least squares (WLSMV) estimation and Crawfer rotation = 0.8, and we extracted 6 factors. We assessed model fit using the comparative fit index (CFI) and root mean squared error of approximation (RMSEA), and we based our model acceptance decisions on cutoff criteria suggested by [5] and [6]. CFI and RMSEA measures of fit estimates were acceptable (see Table S1-3, EF_1_).

We identified 5 constructs of interest based on the patterns of factor-item loadings (Table S1-4a) and factor inter-correlations (Table S1-4b); strong loadings were identified as having an absolute value >.40. For each factor, we selected three indicators to be included in the structural factor analyses of the primary survey data. We labeled these five factors as follows: (a) "Personal Vanity" (indicated by the valuation of power, bravery, and sex appeal as personal attributes), (b) "Family-Building Values" (indicated by desires for marriage, building a house, and children), (c) "Ambition for Higher Education" (indicated by survey items related to desired scholastic achievement), (d) “Town Recreation" (indicated by preferences for dancing/nightclubbing, pool playing, and drinking alcohol as recreational preferences – note, these activities reflect social preferences pursuable in villages but adopted from town), and (e) "Perceived Parental Strictness" (indicated by items asking respondents how they thought their parents would respond upon learning the respondent had a boy/girlfriend, had spent the night out, or were pregnant/had gotten someone pregnant). A sixth factor was primarily reflective of smoking cigarettes as a recreational pursuit and (less so) of the positive valuation of wealth, fashion and trustworthiness and the negative evaluation of having children. The latter three of these items cross-loaded strongly onto other factors, leaving smoking as a unique identifier for the sixth factor, hence our choice to exclude it from the subsequent structural analyses.

We followed a similar approach in factor analyzing the 20 dichotomous items related to spending preferences. Parallel analysis implied that 4 factors could be reasonably extracted from the data for these items. We again used MPLUS's categorical factor analysis software with WLSMV estimation and Crawfer rotation = 0.8 to extract 4 factors. Fit statistics (Table S1-3, EF_2_) were reasonable [5]. Factor-item loadings (Table S1-5a) suggested the following constructs: (a) "personal effects spending", (b) "business spending", (c) "farming investment", and (d) "family requisites". Correlations among the factors were negligible, with the largest being -.15 between the factors of 'farming' and 'family requisites' (Table S1-5b). As before, we selected three indicators per factor for follow-up structural factor analyses of the primary survey data. Items related to the farming investment factor included spending on livestock and farming (positive loadings) as well as spending on education (negative loadings). The educational items loaded more strongly onto this factor than did the farming/livestock items, but we chose to retain the latter for follow-up analyses given our prior selection of ordinal items related to 'educational aspirations'. Pilot survey items related to the family requisites factor included spending on household furnishings and food for family. The primary survey only included two of the three household furnishings items present on the pilot survey, so although household furnishing items were stronger indicators of the family requisites factor, we selected the family food spending items for use in subsequent structural factor models of the primary survey data. We did not perform exploratory factor analysis of the pilot data related to the HIV/AIDS items as parallel analysis favored a 1-factor solution for this category and also for the reason that the research team greatly modified this section on the primary survey.

**Section B. Structural factor analyses of primary survey data**

Based on the outcomes from the exploratory analyses of the pilot study data, we derived two structural factor models. The first model included 15 (ordinal) survey items related to psychographic attributes mapped onto 5 factors (3 items per factor), and the second model included 12 (dichotomous) spending preference items mapped onto 4 factors. We fit each of these models to data from the primary survey using MPLUS statistical software with WLSMV estimation. We again assessed model fit via CFI and RMSEA measures [5]. We constrained factor variances to 1.0 in order to identify the model and for the purpose of factor loading estimation and factor score extraction. Model fit for the psychographic data was acceptable (Table S1-3, SF_1_), and the item loadings for each factor were strong, ranging from .46 to .94 (Table S1-6a). Several of the factors were moderately correlated [7]: Correlations between the family-building values factor and each of the other factors ranged from .25 to .37. Town recreation and perceived parental strictness were positively correlated at .31. See Table S1-6b for additional factor correlation estimates.

Model fit for the spending preference data was poor (Table S1-3, SF_2a_). The software's diagnostic output indicated problems with two of the factors (spending on personal effects and spending on business). We chose to run a second analysis in which we removed the problematic factors and related items from the model. Fit for this 2-factor model (spending of food for family and spending on livestock) was greatly improved (Table S1-3, SF_2b_); however, one of the factor loadings (100k on livestock) was negligible (.02), somewhat surprising given the cost of purchasing a cow often exceeds 800,000 Tanzanian shillings. Factor loadings were strong for all items related to spending on food for family (ranging from .59 to .93; Table S1-6a).

As regards our final predictor variable, HIV/AIDS knowledge, we fit a structural factor model (1 factor) to data from the 10 dichotomous items used to assess such awareness in the primary survey. This analysis was equivalent to an item response theory (IRT) assessment using a 2-parameter probit metric. Model fit was poor (CFI = .63 and RMSEA = .12; Table S1-3, SF_3a_) despite strong factor loadings across the items (all but one were larger than .40). Item discriminations ranged from .29 to .95, and item difficulties ranged from -1.2 to -.40 (Table S1-7), with the most difficult item ("Is it possible to vaccinate against HIV") showing the lowest discriminatory power. Overall, the survey items used to assess HIV knowledge were not sufficiently difficult. Accordingly we chose as indicators of HIV knowledge a subset of 3 items that showed decent discriminatory power while spanning as wide a range of difficulty as possible. Structural factor analysis of these three items showed that their loadings were all strong (ranging from .42 to .72; Table S1-6a). Item difficulties (Table S1-7) ranged from -.75 to -.5.

From the above structural factor analyses, we extracted 7 factor scores for each individual in the primary study: (a) HIV awareness (indicated by responses to three questions related to HIV prevention and transmission: condom use, monogamy, and food sharing), (b) "Personal Vanity" (indicated by the valuation of power, bravery, and sex appeal as personal attributes), (c) "Family-Building Values" (indicated by desires for marriage, building a house, and children), (d) "Ambition for Higher Education" (indicated by survey items related to desired scholastic achievement), (e) “Town Recreation" (indicated by preferences for dancing/nightclubbing, pool playing, and drinking alcohol as leisure preferences – note, these activities reflect social preferences pursuable in villages but adopted from town), and (f) "Perceived Parental Strictness" (indicated by items asking respondents how they thought their parents would react upon learning the respondent had a boy/girlfriend, had spent the night out, or were pregnant/had gotten someone pregnant), and (g) "Prefer Spending on Family" as indicated by the stated willingness to spend on food for family members given low, moderate, and high levels of available funds.

**Section C. Hierarchical regression methodology**

For each of 5 outcome variables (whether an individual had previously been tested for HIV, whether an individual had ever been sexual, whether an individual used a condom when last sexually active, age of first sexual activity, and number of lifetime sexual partners) , we conducted a series of hierarchical regression analyses in which we first fit a baseline (intercept only) model (M0) to the data. We then sequentially added the following sets of predictors and assessed change in model fit: (M1) basic demographic variables (age, gender, highest level of completed education, employment status, Maasai/non-Maasai ethnicity), (M2) village membership (8 villages), (M3) average weekly media consumption (television, radio, print media), (M4) HIV knowledge, (M5) psychographic features (personal vanity, family values, educational ambition, town leisure, perceived parental strictness, and concern for family as indicated by the stated willingness to spend on food for family members) . We used this order to sequentially add less-investigated predictors to more conventional ones.

We added village membership separately from other demographic variables to determine whether specific location might account for observed variance in the outcome measures, analogous to a random effects model. For all analyses, we assessed the change in model fit via log-likelihood ratio testing (e.g., change in deviance, or -2*log-likelihood, relative to change in degrees of freedom) [8]. When addition of a given set of predictors resulted in a significantly lower deviance score, we carried that set forward to the next model. When there was no improvement in fit, we dropped that set of predictors from the model and moved forward to the next set in the sequence. For the three dichotomous outcomes, we also obtained pseudo-R-square values [9] for the best-fitting models. We used Mplus statistical software [4] with full information maximum likelihood (FIML) estimation for all regression analyses (i.e., logistic, Poisson, and survival analyses).

**Section D. Tables**

Table A

Pilot Study Participant Demographics

| Statistic | Value | |
| --- | --- | --- |
| N | 422 | |
| Number of Villages | 6 | |
| % Female | 47 | |
| Mean Age (min, max) | 16.4 (12, 26) | |
| Highest Education Completed |  | |
| % None | 5 | |
| % Primary School | 72 | |
| % Secondary School | 23 | |
| Employment Status |  | |
| % Unemployed | 89^a^ | |
| % Farming |  | |
| % Off-Farm Work ^b^ | 11 | |
|  | female | male |
| % Tested for HIV ^c^ | 20.1 | 24.0 |
| % Non-Virgins | 17.7 | 40.0 |
| % Used a Condom During Last Sex ^d^ | NA | NA |
| Mean (SD) Age at First Sex ^d^ | 16.8 (2.2) | 14.9 (2.8) |
| Mean (SD) # Lifetime Sexual Partners ^d^ | 1.7 (1.2) | 2.4 (2.1) |

^a^ Combined unemployed and farm labor in pilot survey

^b^ Almost invariably this is in addition to farming

^c^ Non-responders were included in calculation of percentage as 'untested'

^d^ Only those who reported being sexually active were included in calculation of percentage

Table B

*Parallel Analyses of Pilot Survey Data*

| Analysis by Category | # Items | # Factors |
| --- | --- | --- |
| Ordinal Items | | |
| 1^st^-pass: analyses by category |  |  |
| Purposes for Travel to Town | 11 | 2 |
| Social and Recreational Activities | 16 | 4 |
| Goals and Aspirations | 12 | 3 |
| Valued Personal Characteristics | 15 | 3 |
| Parental Strictness | 4 | 1 |
| Views and Beliefs | 13 | 2 |
| 2^nd^-pass: analysis of select, pooled items | 36 | 6 |
| Dichotomous Items | | |
| HIV Knowledge | 7 | 1 |
| Spending Preferences | 20 | 4 |

*Note*. # Factors refers to the maximum number of factors deemed extractable via parallel analysis.

Table C

*Model Descriptions and Goodness-of-Fit Statistics for Factor Analyses*

| Model | Description | | *χ*^2^ | *df* | | CFI | | RMSEA (.95 CI) |
| --- | --- | --- | --- | --- | --- | --- | --- | --- |
| *Pilot Survey* | | | | | | | | |
| EF_1_ | Psychographic Attributes (36 items) | 905 | | 429 | .94 | | .05 (.05, .06) | |
| EF_2_ | Spending Preferences | 268 | | 116 | .89 | | .05 (.04, .06) | |
| *Primary Survey* | | | | | | | | |
| SF_1_ | Psychographic Attributes | 275 | | 80 | .94 | | .06 (.05, .07) | |
| SF_2a_ | Spending Preferences (4 Factors) | 456 | | 48 | .69 | | .13 (.12, .14) | |
| SF_2b_ | Spending Preferences (2 Factors) | 24 | | 8 | .92 | | .06 (.03, .09) | |
| SF_3a_ | HIV/AIDS Knowledge (10 items) | 301 | | 35 | .63 | | .12 (.11, .13) | |
| SF_3b_ | HIV/AIDS Knowledge (3 items) | *saturated model* | | | | | | |

*Note*. CFI = comparative fit index; RMSEA = Root Mean Square Error of Approximation; EF = exploratory factor analysis; SF = structural analysis.

Table D

Select Psychographic Items from Pilot Survey: Item-Factor Loadings from EF_1_:

|  | Loadings Each Factor | | | | | |
| --- | --- | --- | --- | --- | --- | --- |
| Survey Item | 1 | 2 | 3 | 4 | 5 | 6 |
| Valued Traits: Bravery | **.78*** | -.21 | .08 | -.01 | .28 | -.13 |
| Valued Traits: Power | **.70*** | -.11 | -.09 | .09 | .12 | .09 |
| Valued Traits: Sex Appeal | **.60*** | .07 | -.32 | .24 | -.32 | -.14 |
| Goals: Have Children | .16 | **.79*** | -.11 | .07 | .25 | -.46 |
| Goals: Get Married | .05 | **.76*** | -.03 | .10 | -.03 | -.22 |
| Goals: Build House | .18 | **.60*** | .20 | -.06 | .05 | -.04 |
| Goals: Finish Higher Degree | .26 | .08 | **.64*** | -.17 | -.29 | -.20 |
| Goals: Finish Secondary School | .19 | .01 | **.63*** | -.19 | -.34 | -.20 |
| Goals: Become a Professional | .35 | .13 | **.58*** | -.12 | -.13 | -.14 |
| Social Activities: Dance (Club/Bar) | .12 | .10 | -.02 | **.65*** | .15 | .20 |
| Social Activities: Play Pool | .03 | -.12 | .41 | **.61*** | .13 | .29 |
| Social Activities: Drink Alcohol (Bar) | .08 | -.08 | -.20 | **.48*** | .25 | .35 |
| Parental Strictness: Pregnancy | -.12 | .09 | -.13 | .12 | **.88*** | .04 |
| Parental Strictness: Boy/Girlfriend | .08 | .16 | .02 | .21 | **.86*** | .01 |
| Parental Strictness: Spent Night Out | -.16 | -.18 | -.09 | .14 | **.65*** | .05 |
| Valued Traits: Personal Beauty | .59 | -.03 | -.39 | .01 | -.28 | -.17 |
| Valued Traits: Athleticism | .57 | -.22 | .24 | -.02 | .09 | -.15 |
| Valued Traits: Fashionableness | .46 | .31 | -.02 | -.04 | -.15 | .45 |
| Valued Traits: Wealth | .42 | .22 | .04 | -.01 | .10 | .46 |
| Goals: Open Business | .06 | .53 | -.21 | .16 | .09 | -.08 |
| Valued Traits: Friendliness | .14 | .44 | .23 | -.09 | -.32 | .32 |
| Valued Traits: Helpfulness | .13 | .43 | .18 | -.02 | -.42 | .21 |
| Valued Traits: Industriousness | .17 | .42 | .23 | -.02 | -.32 | .33 |
| Valued Traits: Trustworthiness | .13 | .41 | .34 | .01 | -.29 | .53 |
| Social Activities: Play Sports Informal | .03 | -.06 | .65 | .29 | -.01 | -.23 |
| Social Activities: Study | -.02 | -.01 | .58 | .08 | -.07 | -.31 |
| Social Activities: Sing in Choir | .01 | -.12 | .43 | .22 | -.22 | -.08 |
| Social Activities: Hang out | .03 | -.12 | .41 | .61 | .13 | .29 |
| Social Activities: Smoke Cigarettes | .20 | .02 | -.19 | .34 | .03 | .99 |
| Goals: Become Wealthy | .24 | .34 | .23 | -.26 | .24 | .45 |
| Social Activities: Conduct Business | .01 | .06 | -.06 | .56 | .02 | -.07 |
| Social Activities: Attend Movies | .05 | .13 | .11 | .50 | -.11 | -.01 |
| Social Activities: Talk with Friends | -.02 | .21 | .14 | .48 | -.08 | -.07 |
| Social Activities: Visit Marketplace | .02 | .06 | -.09 | .45 | -.32 | .09 |
| Social Activities: Farm/Garden | -.10 | -.05 | .09 | .45 | .01 | -.08 |
| Goals: Live in the City | .27 | .39 | .34 | -.33 | .01 | .28 |

Note. Factor loadings shown in bold with an asterisk (*) correspond to items chosen for inclusion in subsequent structural factor analyses.

Table E

Factor Correlation Matrix from EF_1_: Psychographic Features from Pilot Survey

| **Factor** | | **1** | **2** | **3** | **4** | **5** | **6** |
| --- | --- | --- | --- | --- | --- | --- | --- |
| **1** | Personal Vanity |  |  |  |  |  |  |
| **2** | Family-Building Values | .25 |  |  |  |  |  |
| **3** | Ambition for Higher Education | .16 | .17 |  |  |  |  |
| **4** | Town Recreation | .17 | .17 | -.05 |  |  |  |
| **5** | Perceived Parental Strictness | .03 | .01 | -.05 | .14 |  |  |
| **6** | Other | -.14 | -.13 | -.26 | -.01 | .14 |  |

Table F

Spending Preference Items from Pilot Survey: Item-Factor Loadings from EF_2_

|  | Loadings Each Factor | | | |
| --- | --- | --- | --- | --- |
| Survey Item | 1 | 2 | 3 | 4 |
| Clothing/Shoes for Self 10k | **.99*** | .19 | .14 | -.04 |
| Clothing/Shoes for Self 100k | **.59*** | -.27 | -.23 | .14 |
| Clothing/Shoes for Self 50k | **.57*** | -.32 | -.05 | -.20 |
| Business 50k | -.08 | **.98*** | .10 | .05 |
| Business 100k | -.01 | **.88*** | .13 | .06 |
| Business 10k | -.52 | **.71*** | -.15 | -.38 |
| Livestock 50k | -.25 | -.47 | **.71*** | -.19 |
| Livestock 100k | -.06 | -.38 | **.43*** | -.08 |
| Livestock 10k | -.36 | -.24 | **.42*** | .02 |
| Food For Family 100k | .06 | -.04 | -.09 | **.64*** |
| Food For Family 50k | .13 | -.12 | .06 | **.44*** |
| Food For Family 10k | -.38 | .05 | .30 | **.19*** |
| Education 100k | .07 | -.17 | -.83 | .10 |
| Education 10k | -.37 | -.38 | -.52 | .29 |
| Education 50k | -.05 | -.17 | -.86 | -.12 |
| Farm 100k | -.02 | -.02 | .45 | -.22 |
| Home Furnishings 50k | -.07 | -.02 | .02 | .99 |
| Home Furnishings 10k | -.10 | .24 | -.06 | .69 |
| Home Furnishings 100k | -.08 | -.01 | -.09 | .62 |
| Farm 50k | -.01 | -.01 | .16 | -.06 |

Note. Factor loadings shown in bold with an asterisk (*) correspond to items chosen for inclusion in subsequent structural factor analyses.

Table G

Factor Correlation Matrix from EF_2_: Spending Preferences

| Factor | | 1 | 2 | 3 | 4 |
| --- | --- | --- | --- | --- | --- |
| 1 | Personal Effects |  |  |  |  |
| 2 | Business | -.11 |  |  |  |
| 3 | Farming | -.09 | .03 |  |  |
| 4 | Family Requisites | -.03 | -.02 | -.15 |  |

Table H

Item-Factor Loadings from Structural Factor Analyses of Primary Survey Data

| Factor | Loadings by Item | | | |
| --- | --- | --- | --- | --- |
| *Psychographic Attributes* (*Analysis* *SF_1_*) | | | | |
| Personal Vanity | | Power | Sex Appeal | Bravery |
|  | | .85 (.04) | .69 (.04) | .67 (.03) |
| Family-Building Values | | Get Married | Have Children | Build House |
|  | | .79 (.04) | .75 (.04) | .69 (.04) |
| Ambition for Higher Edu. | | Higher Degree | Secondary School | Professional |
|  | | .94 (.02) | .86 (.03) | .78 (.03) |
| Town Recreation | | Play Pool | Drink Alcohol | Dance (Club/Bar) |
|  | | .88 (.21) | .51 (.18) | .46 (.18) |
| Perceived Parental Strictness | | Boy/Girlfriend | Pregnancy | Slept Out |
|  | | .91 (.05) | .79 (.04) | .59 (.04) |
| *Spending Preferences* (*Analysis* *SF_2b_*) | | | | |
| Food For Family | | 10k | 50k | 100k |
|  | | .93 (.08) | .59 (.07) | .65 (.08) |
| Livestock | | 10k | 50k | 100k |
|  | | .72 (.25) | .78 (.24) | .02 (.09) |
| *HIV Knowledge* (*Analysis* *SF_3b_*) | | | | |
| HIV Knowledge | | One Partner^a^ | Condom Use^b^ | Shared Food^c^ |
|  | | .72 (.13) | .55 (.11) | .42 (.09) |

*Note*. Standard errors for each loading are provided in parentheses.

^a^ Can monogamy reduce risk of HIV contraction?

^b^ Can people contract HIV by sharing food with infected persons?

^c^ Can condom use reduce risk of HIV contraction?

Table I

*Factor Correlation Matrix from SF_1_:* Psychographic Features from Primary Survey

| Factor | | 1 | 2 | 3 | 4 | 5 |
| --- | --- | --- | --- | --- | --- | --- |
| 1 | Personal Vanity |  |  |  |  |  |
| 2 | Family-Building Values | .26 |  |  |  |  |
| 3 | Ambition for Higher Education | .12 | .37 |  |  |  |
| 4 | Town Recreation | .09 | .36 | .13 |  |  |
| 5 | Parental Strictness | -.05 | .25 | -.11 | .31 |  |

*Note*. In a separate analysis (SF_2b_), we found a correlation of -.23 between the two spending preference factors (food for family, livestock).

Table J

Item Response Parameter Estimates for HIV Knowledge Section of Primary Survey

| Item | Discrim. | Difficulty |
| --- | --- | --- |
| *HIV Knowledge, 10 items* (*Analysis SF_3a_*) | | |
| Is HIV/AIDS caused by witchcraft? | .95 (.15) | -1.1 (.13) |
| Can a person contract HIV by sharing food with infected persons? | .71 (.10) | -.45 (.11) |
| Is it possible for a healthy looking person to have HIV? | .70 (.10) | -.44 (.10) |
| Can a baby contract HIV from his/her mother during parturition? | .67 (.09) | -.55 (.11) |
| Can a baby contract HIV from his/her mother via breastfeeding? | .65 (.10) | -.86 (.13) |
| Can people contract HIV via mosquito bite? | .62 (.10) | -.90 (.16) |
| Is it possible for a fetus to contract HIV from an infected mother? | .56 (.09) | -.69 (.14) |
| Can monogamy reduce risk of HIV contraction? | .49 (.09) | -1.2 (.23) |
| Can condom use reduce risk of HIV contraction? | .49 (.09) | -.64 (.16) |
| Is it possible to vaccinate against HIV/AIDS? | .29 (.07) | -.40 (.23) |
| *HIV Knowledge, 3 items* (*Analysis SF_3b_*) | | |
| Can monogamy reduce risk of HIV contraction? | 1.0 (.40) | -.75 (.17) |
| Can people contract HIV by sharing food with infected persons? | .46 (.12) | -.63 (.20) |
| Can condom use reduce risk of HIV contraction? | .66 (.19) | -.51 (.15) |

*Note*. Standard errors for each estimate are provided in parentheses. Item response parameters (discrimination, difficulty) were computed by MPLUS statistical software in a 2-parameter probit metric.

**Section E. Discussion of age, sex, education, employment and ethnicity effects**

Data relevant to this discussion are shown in Tables 2 and 3 of the main article.

We found that older individuals in our sample are not only more likely to be non-virgins, but are also more likely to have been screened for HIV and (among the sexually active) to have had a greater number of lifetime sexual partners. These results parallel the common finding that mid-reproductive career individuals are more likely not to be virgins and to be knowledgeable about the risks of HIV infection - as in the Kilimanjaro region of Tanzania; see [10]. They are also more likely to have multiple lifetime sexual partners, unless there has been a sharp recent change in behavior in this domain - as in some parts of southern Africa; see [11]

Women in our study were more likely than men to be sexually active. This is consistent with the higher HIV prevalence among women than men in countries with widespread HIV/AIDS epidemics [12-15] and the typical earlier onset of HIV infection among women - as shown for Malawi; see [16]. Women’s greater probability of being sexually active than men, even controlling for age, likely reflects age mixing (younger girls with older men), earlier marriage for girls, and gender inequalities in power (most acutely the high unwanted pregnancy rate among schoolgirls, so often attributed to teachers and older pupils). Such dynamics however do not lead to a significantly earlier age at virginity loss among women in this sample. Women in our sample were also more likely to have been screened for HIV and reported fewer lifetime sex partners then men. These are also commonly reported patterns [11], the former most likely reflecting the efforts of maternal child health clinic-based programs, and the latter either a reflection of gender-specific underreporting [17] or a greater tendency for men to seek multiple partners [18] - most likely in this case amongst women living outside the sampled village populations. Women in our sample show similar levels of HIV knowledge as do men (data not shown), despite recent evidence from Aarø et al. [19] suggesting that women in Tanzania, even in the capital, lag far behind men in terms of knowledge about HIV. Perhaps the situation in Tanzania is changing – Aarø et al.’s baseline data comes from 2004. Finally, we note that our HIV knowledge item scale performed poorly, a result similar to that reported by Aarø et al. [19]: Our questions were either too easy or lacked discriminatory power, or both (see Supplemental Digital Content 2), suggesting that as awareness of HIV spreads into rural areas, education needs to become more sophisticated and, incidentally, knowledge scales be redesigned.

Education, particularly secondary education, matters, although the effects of education on infection rates, knowledge about HIV/AIDS, and risky behavior are quite variable [20], most probably reflecting the stage which the epidemic has reached [21]. Educated youth in this rural sample are more likely to have tested for HIV, and are less likely to have engaged in sexual activity at a younger age than their less educated co-villagers, concordant with the evidence from nationally representative samples in Tanzania that HIV/AIDS prevalence declines with education [22] and with qualitative ethnographic resarch [15]. Furthermore those with a secondary education have fewer sexual partners and are more likely to use condoms Nevertheless, and contrary to these patterns, among those who are sexually active, completion only of primary (but not secondary) education is associated with a higher number of lifetime sexual partners; causality here though is uncertain – do students who achieve only a primary school education prefer multiple sexual partners, or do sexually active students fail to pass the exams needed for getting into secondary school? More generally these patterns suggest that expanding secondary school education will do much to slow down the spread of the pandemic in rural areas.

Regarding employment, farming (had no effect on any behavioral outcome, whereas those employed in off-farm enterprises (usually small village-based business like carpentry or retail) reported older ages at which they lost their virginity (off-farm employment was a negative predictor of hazard of virginity loss). This is an encouraging development insofar as such individuals constitute models in village life as people who have successfully diversified their economic portfolio to some extent.

As regards ethnicity, the Maasai are generally the most traditional people living in northern Tanzania on account of their commitment to pastoralism in the drier and more remote areas and their tendency to self-identify as indigenous people; given a tendency among at least some Maasai communities, to resist outside interference, our results are for the most part to be expected. Compared to other ethnicities, Maasai are more likely to be non-virgins and to have initiated sexual activity at a younger age, and more likely to have multiple sexual partners (as reported for both males and females, unpublished data). Rather intriguingly, they are also *more* likely to have been tested for HIV. The greater tendency of Maasai to be tested may reflect the targeting of campaigns in the area, targeting that appears to be ethnic rather than village-based (there is no village level effect). Certainly the Maasai, with their colorful traditional life styles are attractive to NGO involvement, and have been the focus of multiple interventions by religious and other civil society organizations, often headed by expatriates, using educational videos and other kinds of campaign strategies. This may be why our study shows that it is the non Maasai who are least likely to opt for HIV-testing.

Finally, additional findings from the model improvement fit in Table 2 are that village-specific effects in this part of northern Tanzania are important with respect to condom use and number of sexual partners, suggestive of idiosyncratic dynamics that cannot be attributed to standard demographic variables, and consistent with variation among villages. Additionally, and rather surprisingly, the role of differential media consumption, either the kind (TV, radio, magazine, newspapers) or quantity accessed, is not an important predictor of any of our risky sexual behavior outcomes in this rural sample, even though radios, magazines and sometimes TV are somewhat available in most places.

**Section F. References**

1) Widaman KF, Reise SP (1997) Exploring the measurement invariance of psychological instruments: Applications in the substance use domain. In: Bryant KJ, Windle M, West SG, editors. The science of prevention: Methodological advances from alcohol and substance abuse research. Washington, DC: American Psychological Association. pp. 281-324.

2) R Development Core Team (2012) R: A language and environment for statistical computing [computer software]. Vienna, Austria. Available at http://www.R-project.org/. Accessed September, 2012.

3) Revelle W (2012) Psych: Procedures for personality and psychological research [computer software]. Available at http://personality-project.org/r/psych.manual.pdf . Accessed September, 2012.

4) Muthén LK, Muthén BO (1998-2013) Mplus user's guide. 7th ed.. Los Angeles, CA: Muthén & Muthén. 850 p.

5) Bentler PM (1990) Comparative fit indexes in structural models. Psychol Bull 107: 238-246.

6) Chen FN, Curran PJ, Bollen KA, Kirby J, Paxton P (2008) An empirical evaluation of the use of fixed cutoff points in RMSEA test statistic in structural equation models. Sociol Methods Res 36: 462-494.

7) Cohen J (1988) Statistical power analysis for the behavioral sciences. 2nd ed. NJ: Lawrence Erlbaum.

8) Wilks SS (1938) The large-sample distribution of the likelihood ratio for testing composite hypotheses. Ann Math Stat 9: 60-62.

9) McKelvey RD, Zavoina W (1975) A statistical model for the analysis of ordinal level dependent variables. J Math Soc 4:103-120.

10) Mmbaga EJ, Leyna GH, Mnyika KS, Klepp K-I (2008) Sexually transmitted infections knowledge and its impact on the practice of risky sexual behaviors and HIV serostatus: Results from rural Kilimanjaro, Tanzania. Sex Transm Infect 84: 224-226.

11) Todd J, Cremin I, McGrath N, Bwanika JB, Wringe A, et al. (2009) Reported number of sexual partners: Comparison of data from four African longitudinal studies. Sex Transm Infect. 85(Suppl. 1): i72-i80.

12) Laga M, Schwartlander B, Pisani E, Sow PS, Carael M (2001) To stem HIV in Africa, prevent transmission to young women. AIDS 15: 931-934.

13) Pettifor A, Rees H, Kleinschmidt I, Steffenson A, MacPhail C, et al. (2012) HIV and sexual behavior among young South Africans: A national survey of 15-24 year olds. Johannesburg, South Africa: Reproductive Health Research Unit, University of the Witwatersrand. Available at http://www.kff.org/southafrica/loader.cfm?url=/commonspot/security/getfile.cfm&PageID=34051. Created 2004; Accessed September, 2012.

14) Stirling M, Rees H, Kasedde S, Hankins C (2008) Introduction: Addresing the vulnerability of young women and girls to stop the HIV epidemic in southern Africa. AIDS 22: S1-S3.

15) Plummer ML, Wight D (2011) Young people's lives and sexual relationships in rural Africa: Findings from a large qualitative study in Tanzania. Lanham: Lexington Books. 464 p.

16) Clark S, Poulin M, Kohler H-P (2009) Marital aspirations, sexual behaviors, and HIV/AIDS in rural Malawi. J Marriage Fam 71: 396-416.

17) Lindstrom DP, Belachew T, Hadley C, Hattori MK, Hogan D, et al. (2010) Nonmarital sex and condom knowledge among Ethiopian young people: Improved estimates using a nonverbal response card. Stud Fam Plann 41: 251-262.

18) Courtiol A, Pettay JE, Jokela M, Rotkirch A, Lummaa V (2012) Natural and sexual selection in a monogamous historical human population. PNAS 109: 8044-8049.

19) Aarø LE, Breivik K, Klepp KI, Kaaya S, Onya HE, et al. (2011) An HIV/AIDS knowledge scale for adolescents: Item response theory analyses based on data from a study in South Africa and Tanzania. Health Educ Res 26: 212-224.

20) Strauss J, Thomas D (2007) Health over the life course. In: Schultz TP, Strauss J, editors. Handbook of development economics. Amsterdam: Elsevier/North-Holland. pp. 3375-3474.

21) Iorio D, Santaeulàlia-Llopis R (2011) Education, HIV status, and risky sexual behavior: How much does the stage of the HIV epidemic matter? Available at http://rsantaeulalia.wustl.edu/pdfs/DHS17.pdf. Accessed September, 2012.

22) Hargreaves JR, Howe LD (2010) Changes in HIV prevalence among differently educated groups in Tanzania between 2003 and 2007. AIDS 24: 755-761.
