# Supplementary material for: Attitudinal and Behavioral Characteristics Predict High Risk Sexual Activity in Rural Tanzanian Youth
Source: PLoS One. 2014 Jun 13;9(6):e99987. doi: 10.1371/journal.pone.0099987 (PMC4057388; doi:10.1371/journal.pone.0099987)
Supplement: File S2 — Survey administered to Tanzanian youth to assess demographic, attitudinal and behavioral characteristics in relation to HIV/AIDS outcomes. (DOC) [file pone.0099987.s002.doc]

| **Youth Study**  **IDENTIFICATION** | | | | | | | | | | | | | |
| --- | --- | --- | --- | --- | --- | --- | --- | --- | --- | --- | --- | --- | --- |
| Region | | |  | | | | | | | | | | |
| District | | |  | | | | | | | | | | |
| Ward | | |  | | | | | | | | | | |
| Village name | | | Unique identifier | | | | | | | | | | |
| Subvillage name | | |  | | | | | | | | | | |
| Household # | | | Unique identifier | | | | | | | | | | |
| Head of Household | | | Unique identifier | | | | | | | | | | |
| Interviewee  Name | | | Unique identifier | | | | | | | | | | |
| Interviewer  Name | | |  | | | | | | | | | | |
| **INTERVIEWER VISITS** | | | | | | | | | | | | |  |
|  | | | | 1 | | 2 | | 3 |  | | | |  |
| Date Unique identifier | | | | _______ | | _______ | | _______ |  | | |  |  |
| Next visit | Date | | | _______ | | _______ | |  |  | | |  |  |
|  | Time | | | _______ | | _______ | |  | | |  |  |
|  | | | | | | | | |  | | |  |  |
| * | | | | | | | | | | |  | |  |
| Date/Time started | | __________ | | | | | | | | | | |  |
| Time ended | | __________ | |  | | |  | | |  | | |  |
| Supervisor name | | | | |  | | | | | | | |  |

**SECTION A:** BACKGROUND EDUCATION AND SES MATERIAL

| **001.1** | **001.2** | **001.3** | **001.4** | **001.5** | **001.6** | **001.7** | **001.8** | **001.9** | **001.10** |
| --- | --- | --- | --- | --- | --- | --- | --- | --- | --- |
| Age (12-24) | Gender  00 male  01 female | Ethnic Group (SES tribe list) | Have you ever attended school?  00 No SKIP TO 004  01 Yes | Are you currently enrolled in school?  00 No  01 Yes | What is the highest level of education you have attained?  00 Nursery  01 Standard 1  02 Standard 2  03 Standard 3  04 Standard 4  05 Standard 5  06 Standard 6  07 Standard 7  08 form 1  09 form 2  10 form 3  11 form 4  12 form 5  13 form 6  14 Certificate  15 Diploma  16 Vocational  17 Degree/university  18 Adult education  19 other type of education  20 none | IF ANSWER TO Q001.3b is 14-19, THEN ASK: What was the highest standard or form you have attained?  00 Nursery  01 Standard 1  02 Standard 2  03 Standard 3  04 Standard 4  05 Standard 5  06 Standard 6  07 Standard 7  08 form 1  09 form 2  10 form 3  11 form 4  12 form 5  13 form 6 | If still studying, Is the school you are now enrolled a public or private?  01 Public  02 Private | Have you ever repeated a grade of school?  00 No  01 Yes | How many times have you repeated a grade of school?  ________ |

|  | | ****Relationship Codes**  **0. None**   1. Self 2. Father 3. Mother 4. Both Parents 5. Uncle 6. Aunt | | | | | | | 1. Grandparents 2. Brother 3. Sister 4. Spouse 5. Church | | | | | | | | | | | | | | | | | | | | | | 1. Friend 2. Other NGO (list)_________ 3. Other relative (list)________   99. Other 9 ______________ | | | | | | | | | | | | | | | | | | | | | | | | |  |
| --- | --- | --- | --- | --- | --- | --- | --- | --- | --- | --- | --- | --- | --- | --- | --- | --- | --- | --- | --- | --- | --- | --- | --- | --- | --- | --- | --- | --- | --- | --- | --- | --- | --- | --- | --- | --- | --- | --- | --- | --- | --- | --- | --- | --- | --- | --- | --- | --- | --- | --- | --- | --- | --- | --- | --- | --- |
| 002 | | Who pays for the majority of your school fees? | | | | | | **Relationship Code | | | | | | | | | | | | | | | | | | | | | | | | | | | | | | | | | | | | | | | | | | | | | | | |  |
| 002a | | Who else contributes? | | | | | | **Relationship Code(s) | | | | | | | | | | | | | | | | | | | | | | | | | | | | | | | | | | | | | | | | | | | | | | | |  |
| 003 | | How likely is it that you will continue to attend school? | | | | | Definitely will not attend  1 | | | | | | Probably will not attend  2 | | | | | | | Will possibly  attend  3 | | | | | | | | | | | | | Probably will attend  4 | | | | | | | | | | | Will definitely attend  5 | | | | | Don’t know  88 | | | | | | |  |
| 004 | | Do you farm? | | | | | | | | Yes  01 | | | | | | | | | | | | | | | | | | | | | | | | | | No  00  (SKIP TO 005) | | | | | | | | | | | | | | |  | | | |  | |
| 004a | | If yes to qn004, Do you farm for your parents, yourself or others? TICK ALL THAT APPLY | | | | | | | | a for my parents Y/N SKIP TO 005  b for myself Y/N GO TO 004b  c for others Y/N SKIP TO 005 | | | | | | | | | | | | | | | | | | | | | | | | | | | | | | | | | | | | | | | | |  | | | |  | |
| 004b | | The farming you do for yourself, is it subsistence or for cash? (NOTE that if answer is subsistence and cash, we should note cash) | | | | | | | | 01 Subsistence | | | | | | | | | | | | | | | | | | | | | | | | | | 02 Cash | | | | | | | | | | | | | | |  | | | |  | |
| 005 | | Do you have any off-farm employment? | | | | | | | | Yes  01 | | | | | | | | | | | | | | | | | | | | | | | | | | No  00  (SKIP TO SECTION B) | | | | | | | | | | | | | | |  | | | |  | |
| 005a | | What type of job do you have? (Just main job. Do not read answers) | | | | | | | | 1. Selling goods 2. Casual labor | | | | | | | | | | | | | | | | | | | | | | 1. Farming (paid labor) 2. Fishing 3. Beekeeping   99 Other (Specify)  88 DK  77 NA | | | | | | | | | | | | | | | | | | | | | | |  | |
| 005b | | Which best describes your current off-farm employment? (Read all answers out loud) | | | | | | | | 1. Full-time:  ( 30 hours a week or more) | | | | | | | | | | | | | | 2. Part-time  (a steady job but less than 30 hours per week) | | | | | | | | | | | | | | | | 3. Casual labor (Temporary jobs when work is available) | | | | | | | | | | | | | | |  | |
| **SECTION B:** TRAVEL PREFERENCES | | | | | | | | | | | | | | | | | | | | | | | | | | | | | | | | | | | | | | | | | | | | | | | | | | | | | | | |  |
| 001 | | How often do you usually visit your district town in a month? | | | | | 1 =Never SKIP TO 003  2 =less than once a month  3=twice a month  4=3 times a month  5=more | | | | | | | | | | | | | | | | | | | | | | | | | | | | | | | | | | | | | | | | | | | | | | | | |  |
| 002 | | How often do you engage in the following activities when you are in a district town? (Read all activities and the scale out loud) | | | | | | | | | | | | | | | | | | | | | | | | | | | | | | | | | | | | | | | | | | | | | | | | | | | | | |  |
| 002a | | Conduct business | | | | Never  1 | | | | | Rarely  2 | | | | | | | Sometimes  3 | | | | | | | | | | | | Often  4 | | | | | | | | | | | | Very Often  5 | | | | | | | | | | | | | |  |
| 002b | | Visit or hang out with friends | | | | | Never  1 | | | | | | Rarely  2 | | | | | | | Sometimes  3 | | | | | | | | | | | | | Often  4 | | | | | | | | | | | Very Often  5 | | | | | | | | | | | |  |
| 002c | | Go to the market | | | | | Never  1 | | | | | | Rarely  2 | | | | | | | Sometimes  3 | | | | | | | | | | | | | Often  4 | | | | | | | | | | | Very Often  5 | | | | | | | | | | | |  |
| 002d | | Participate in festivals or special events | | | | | Never  1 | | | | | | Rarely  2 | | | | | | | Sometimes  3 | | | | | | | | | | | | | Often  4 | | | | | | | | | | | Very Often  5 | | | | | | | | | | | |  |
| 002e | | Attend school (formal education) | | | | | Never  1 | | | | | | Rarely  2 | | | | | | | Sometimes  3 | | | | | | | | | | | | | Often  4 | | | | | | | | | | | Very Often  5 | | | | | | | | | | | |  |
| 002f | | Attend vocational or trade school | | | | | Never  1 | | | | | | Rarely  2 | | | | | | | Sometimes  3 | | | | | | | | | | | | | Often  4 | | | | | | | | | | | Very Often  5 | | | | | | | | | | | |  |
| 002g | | Go to a dance club or bar | | | | | Never  1 | | | | | | Rarely  2 | | | | | | | Sometimes  3 | | | | | | | | | | | | | Often  4 | | | | | | | | | | | Very Often  5 | | | | | | | | | | | |  |
| 002h | | Watch videos or television | | | | | Never  1 | | | | | | Rarely  2 | | | | | | | Sometimes  3 | | | | | | | | | | | | | Often  4 | | | | | | | | | | | Very Often  5 | | | | | | | | | | | |  |
| 002i | | Visit the doctor or hospital | | | | | Never  1 | | | | | | Rarely  2 | | | | | | | Sometimes  3 | | | | | | | | | | | | | Often  4 | | | | | | | | | | | Very Often  5 | | | | | | | | | | | |  |
| 002j | | Attend seminars or workshops | | | | | Never  1 | | | | | | Rarely  2 | | | | | | | Sometimes  3 | | | | | | | | | | | | | Often  4 | | | | | | | | | | | Very Often  5 | | | | | | | | | | | |  |
| 003 | | What is the farthest place you have ever been? (READ LIST) | | | | | 01 Same village  02 Same ward  03 Same District  04 Same Region  05 Same Country/another region  06 Another country | | | | | | | | | | | | | | | | | | | | | | | | | | | | | | | | | | | | | | | | | | | | | | | | |  |
| 004 | | We would like to know about your ideas for the future. Please rate how likely you think it is that you will move to a different location in the future. I am going to read a list of possible places you could live in the future and I would like you to say whether it is it (read list of answers) that you will live in that location. | | | | | | | | | | | | | | | | | | | | | | | | | | | | | | | | | | | | | | | | | | | | | | | | | | | | | |  |
| 004a | | Where are you most likely to live in the future? (READ LIST) | | | | | 01 Same village  02 Same ward  03 Same District  04 Same Region  05 Same Country/another region  06 Another country | | | | | | | | | | | | | | | | | | | | | | | | | | | | | | | | | | | | | | | | | | | | | | | | |  |
| 004b | | Why would you like to live there? (Tick the one most highly rated) | | | | | Family lives there  Job prospects are better  A pretty area  Education opportunities  Other (list)_________________________________  DK  NA | | | | | | | | | | | | | | | | | | | | | | | | | | | | | | | | | | | | | | | 1  2  3  4  99  88  77 | | | | | | | | | |  |
| 005 | | Who do you live with? | | | | | **Relationship Codes**   1. Self 2. Father (birth/biological father) 3. Mother (birth/ biological mother) 4. Both biological Parents 5. Uncle 6. Aunt 7. Grandparents   8. Brother (younger or older) | | | | | | | | | | | | | | | | | | | | 9. Sister (younger or older)  10. Spouse  11. Church  12. Friend  13. NGO (list)_________  14. Other relative (list)____________  99 Other  88 DK  77 NA | | | | | | | | | | | | | | | | | | | | | | | | | | | | |  |
| 005a | | If not with your parents; Why are you living apart from your parents? | | | | | Attending school  Parents died  Parent divorce or separated  Parent remarried and left behind  Other (list)________  DK  NA | | | | | | | | | | | | | | | | | | | | 1  2  3  4  99  88  77 | | | | | | | | | | | | | | | | | | | | | | | | | | | | |  |
| 006 | | What religion, if any, do you practice? | | | | | 01 Muslim | | | | | 02 Catholic | | | | | | | 03 Protestant | | | | | | | | | | | | | | | 04 Tradition | | | | | | | | | 05 None | | | | | 99 Other | | | | | | | |  |
| 006a | | How often do you attend services? | | | | | 1=Never  2=Less than once a month  3=Once a month  4=Two or three times a month  5=Every week | | | | | | | | | | | | | | | | | | | | | | | | | | | | | | | | | | | | | | | | | | | | | | | | |  |
| 006b | | Do you participate in additional church/mosque or traditional religion activities? | | | | | Yes 01 | | | | | | | | | | | | | | | | | | | | | No 00  GO TO 008 | | | | | | | | | | | | | | | | | | | | | | | | | | | |  |
| 006c | | Which ones? (Circle all that apply) | | | | | a. Choir  b. Cleaning  c. Participate in services  d. Participate in special ceremonies  e. Education programs (Bible study, Islamic equivalent)  f. Read scriptures  g. Ministry  h. Church elder, usher  i. Teaching others children/elders  j. Other (list)__________  k. DK  l. NA | | | | | | | | | | | | | | | | | | | | | | | | | | | | | | | | | | | | | | | | | | | | | | | | |  |
| 007 | | How frequently do you and your friends engage in the following activities? | | | | | Never  1 | | | | | | | | Rarely  2 | | | | | | | | Occasionally  3 | | | | | | | | | | | | Often  4 | | | | | | | | | | Very  Often  5 | | | | | | | | | | |  |
| 007a | | Walk around together | | | | | Never  1 | | | | | | | | Rarely  2 | | | | | | | | Occasionally  3 | | | | | | | | | | | | Often  4 | | | | | | | | | | Very  Often  5 | | | | | | | | | | |  |
| 007b | | Talk together about our lives | | | | | Never  1 | | | | | | | | Rarely  2 | | | | | | | | Occasionally  3 | | | | | | | | | | | | Often  4 | | | | | | | | | | Very  Often  5 | | | | | | | | | | |  |
| 007c | | Work together outside of school | | | | | Never  1 | | | | | | | | Rarely  2 | | | | | | | | Occasionally  3 | | | | | | | | | | | | Often  4 | | | | | | | | | | Very  Often  5 | | | | | | | | | | |  |
| 007d | | Choir | | | | | Never  1 | | | | | | | | Rarely  2 | | | | | | | | Occasionally  3 | | | | | | | | | | | | Often  4 | | | | | | | | | | Very  Often  5 | | | | | | | | | | |  |
| 007e | | Play sports on a formal team | | | | | Never  1 | | | | | | | | Rarely  2 | | | | | | | | Occasionally  3 | | | | | | | | | | | | Often  4 | | | | | | | | | | Very  Often  5 | | | | | | | | | | |  |
| 007f | | Play sports informally | | | | | Never  1 | | | | | | | | Rarely  2 | | | | | | | | Occasionally  3 | | | | | | | | | | | | Often  4 | | | | | | | | | | Very  Often  5 | | | | | | | | | | |  |
| 007g | | Study | | | | | Never  1 | | | | | | | | Rarely  2 | | | | | | | | Occasionally  3 | | | | | | | | | | | | Often  4 | | | | | | | | | | Very  Often  5 | | | | | | | | | | |  |
| 007h | | Do business together | | | | | Never  1 | | | | | | | | Rarely  2 | | | | | | | | Occasionally  3 | | | | | | | | | | | | Often  4 | | | | | | | | | | Very  Often  5 | | | | | | | | | | |  |
| 007i | | Farm or Garden | | | | | Never  1 | | | | | | | | Rarely  2 | | | | | | | | Occasionally  3 | | | | | | | | | | | | Often  4 | | | | | | | | | | Very  Often  5 | | | | | | | | | | |  |
| 007j | | Go to the market | | | | | Never  1 | | | | | | | | Rarely  2 | | | | | | | | Occasionally  3 | | | | | | | | | | | | Often  4 | | | | | | | | | | Very  Often  5 | | | | | | | | | | |  |
| 007k | | Visit dance clubs or bars | | | | | Never  1 | | | | | | | | Rarely  2 | | | | | | | | Occasionally  3 | | | | | | | | | | | | Often  4 | | | | | | | | | | Very  Often  5 | | | | | | | | | | |  |
| 007l | | Play pool | | | | | Never  1 | | | | | | | | Rarely  2 | | | | | | | | Occasionally  3 | | | | | | | | | | | | Often  4 | | | | | | | | | | Very  Often  5 | | | | | | | | | | |  |
| 007m | | Watch Movies or Television | | | | | Never  1 | | | | | | | | Rarely  2 | | | | | | | | Occasionally  3 | | | | | | | | | | | | Often  4 | | | | | | | | | | Very  Often  5 | | | | | | | | | | |  |
| 007n | | Drink Alcoholic  Beverages  Refused 66 | | | | | Never  1 | | | | | | | | Rarely  2 | | | | | | | | Occasionally  3 | | | | | | | | | | | | Often  4 | | | | | | | | | | Very Often  5 | | | | | | | | | | |  |
| 007o | | Smoke Cigarettes  Refused 66 | | | | | Never  1 | | | | | | | | Rarely  2 | | | | | | | | Occasionally  3 | | | | | | | | | | | | Often  4 | | | | | | | | | | Very Often  5 | | | | | | | | | | |  |
| 007p | | Use other drugs  Refused 66 | | | | | Never  1 | | | | | | | | Rarely  2 | | | | | | | | Occasionally  3 | | | | | | | | | | | | Often  4 | | | | | | | | | | Very Often  5 | | | | | | | | | | |  |
| 007q | | Other | | | | | Never  1 | | | | | | | | Rarely  2 | | | | | | | | Occasionally  3 | | | | | | | | | | | | Often  4 | | | | | | | | | | Very Often  5 | | | | | | | | | | |  |
| SECTION C: MEDIA AND OTHER INFLUENCES | | | | | | | | | | | | | | | | | | | | | | | | | | | | | | | | | | | | | | | | | | | | | | | | | | | | | | | |  |
| 001 | | Do you watch television or videos? | | | | | 00 No SKIP TO 003  01 Yes | | | | | | | | | | | | | | | | | | | | | | | | | | | | | | | | | | | | | | | | | | | | | | | | |  |
| 001a | | Where do you watch television or movies most often? | | | | | At home with family only  Private homes with a friend  Private homes in groups of more than 2  Local guest houses or bars  Other  DK  NA | | | | | | | | | | | | | | | | | | | | | | | | | | | | | | | | | | | | | | | 1  2  3  4  99  88  77 | | | | | | | | | |  |
| 002 | | How often do you watch? | | | | | 1=never  2=rarely  3=occasionally  4=often  5=very often. | | | | | | | | | | | | | | | | | | | | | | | | | | | | | | | | | | | | | | | 1  2  3  4  5 | | | | | | | | | |  |
| 002a | | Comedy (Original comedy, futuhi, ze comedy, Mizengwe e.t.c) | | | | | Never  1 | | | | | | | | Rarely  2 | | | | | | | | Occasionally  3 | | | | | | | | | | | | Often  4 | | | | | | | | | | Very  Often  5 | | | | | | | | | | |  |
| 002b | | Sports (soccer, wrestling, basketball, boxing e.t.c) | | | | | Never  1 | | | | | | | | Rarely  2 | | | | | | | | Occasionally  3 | | | | | | | | | | | | Often  4 | | | | | | | | | | Very  Often  5 | | | | | | | | | | |  |
| 002c | | News | | | | | Never  1 | | | | | | | | Rarely  2 | | | | | | | | Occasionally  3 | | | | | | | | | | | | Often  4 | | | | | | | | | | Very  Often  5 | | | | | | | | | | |  |
| 002d | | Gospel music/preaching | | | | | Never  1 | | | | | | | | Rarely  2 | | | | | | | | Occasionally  3 | | | | | | | | | | | | Often  4 | | | | | | | | | | Very  Often  5 | | | | | | | | | | |  |
| 002e | | Worldly music (Non gospel music e.g ) | | | | | Never  1 | | | | | | | | Rarely  2 | | | | | | | | Occasionally  3 | | | | | | | | | | | | Often  4 | | | | | | | | | | Very  Often  5 | | | | | | | | | | |  |
| 002f | | TV dramas/soap operas/movies | | | | | Never  1 | | | | | | | | Rarely  2 | | | | | | | | Occasionally  3 | | | | | | | | | | | | Often  4 | | | | | | | | | | Very  Often  5 | | | | | | | | | | |  |
| 002g | | Reality shows (Uswazi, Rooving DJ, my wedding, chereko e.t.c) | | | | | Never  1 | | | | | | | | Rarely  2 | | | | | | | | Occasionally  3 | | | | | | | | | | | | Often  4 | | | | | | | | | | Very  Often  5 | | | | | | | | | | |  |
| 002h | | Educational Programs e. g debates, environmental education | | | | | Never  1 | | | | | | | | Rarely  2 | | | | | | | | Occasionally  3 | | | | | | | | | | | | Often  4 | | | | | | | | | | Very  Often  5 | | | | | | | | | | |  |
| 002i | | TV debates | | | | | Never  1 | | | | | | | | Rarely  2 | | | | | | | | Occasionally  3 | | | | | | | | | | | | Often  4 | | | | | | | | | | Very  Often  5 | | | | | | | | | | |  |
| 002j | | Others | | | | | Never  1 | | | | | | | | Rarely  2 | | | | | | | | Occasionally  3 | | | | | | | | | | | | Often  4 | | | | | | | | | | Very  Often  5 | | | | | | | | | | |  |
| 003 | | Do you listen to the radio? | | | | | **00 No SKIP TO 005**  01 Yes | | | | | | | | | | | | | | | | | | | | | | | | | | | | | | | | | | | | | | | | | | | | | | | | |  |
| 004 | | How often do you listen to___? | | | | | 1=never  2=rarely  3=occasionally  4=often  5=very often. | | | | | | | | | | | | | | | | | | | | | | | | | | | | | | | | | | | | | | | 1  2  3  4  5 | | | | | | | | | |  |
| 004a | | Comedy | | | | | Never  1 | | | | | | | | Rarely  2 | | | | | | | | Occasionally  3 | | | | | | | | | | | | Often  4 | | | | | | | | | | Very  Often  5 | | | | | | | | | | |  |
| 004b | | Narrative stories | | | | | Never  1 | | | | | | | | Rarely  2 | | | | | | | | Occasionally  3 | | | | | | | | | | | | Often  4 | | | | | | | | | | Very  Often  5 | | | | | | | | | | |  |
| 004c | | Sports | | | | | Never  1 | | | | | | | | Rarely  2 | | | | | | | | Occasionally  3 | | | | | | | | | | | | Often  4 | | | | | | | | | | Very  Often  5 | | | | | | | | | | |  |
| 004d | | News | | | | | Never  1 | | | | | | | | Rarely  2 | | | | | | | | Occasionally  3 | | | | | | | | | | | | Often  4 | | | | | | | | | | Very  Often  5 | | | | | | | | | | |  |
| 004e | | Gospel music/preaching | | | | | Never  1 | | | | | | | | Rarely  2 | | | | | | | | Occasionally  3 | | | | | | | | | | | | Often  4 | | | | | | | | | | Very  Often  5 | | | | | | | | | | |  |
| 004f | | Worldly music (Non gospel music e.g ) | | | | | Never  1 | | | | | | | | Rarely  2 | | | | | | | | Occasionally  3 | | | | | | | | | | | | Often  4 | | | | | | | | | | Very  Often  5 | | | | | | | | | | |  |
| 004g | | Dramas/soap operas | | | | | Never  1 | | | | | | | | Rarely  2 | | | | | | | | Occasionally  3 | | | | | | | | | | | | Often  4 | | | | | | | | | | Very  Often  5 | | | | | | | | | | |  |
| 004h | | Reality programs (maisha, mikasa, maisha ya watu) | | | | | Never  1 | | | | | | | | Rarely  2 | | | | | | | | Occasionally  3 | | | | | | | | | | | | Often  4 | | | | | | | | | | Very  Often  5 | | | | | | | | | | |  |
| 004i | | Educational Programs e. g debates, environmental education | | | | | Never  1 | | | | | | | | Rarely  2 | | | | | | | | Occasionally  3 | | | | | | | | | | | | Often  4 | | | | | | | | | | Very  Often  5 | | | | | | | | | | |  |
| 004j | | Others | | | | | Never  1 | | | | | | | | Rarely  2 | | | | | | | | Occasionally  3 | | | | | | | | | | | | Often  4 | | | | | | | | | | Very  Often  5 | | | | | | | | | | |  |
| 005 | | How often do you read the following types of magazines__? | | | | | 1=never  2=rarely  3=occasionally  4=often  5=very often. | | | | | | | | | | | | | | | | | | | | | | | | | | | | | | | | | | | | | | | | | | | | | | | | |  |
| 005a | | FEMINA | | | | | Never  1 | | | | | | | | Rarely  2 | | | | | | | | Occasionally  3 | | | | | | | | | | | | Often  4 | | | | | | | | | | Very  Often  5 | | | | | | | | | | |  |
| 005b | | Bongo | | | | | Never  1 | | | | | | | | Rarely  2 | | | | | | | | Occasionally  3 | | | | | | | | | | | | Often  4 | | | | | | | | | | Very  Often  5 | | | | | | | | | | |  |
| 005c | | Sani | | | | | Never  1 | | | | | | | | Rarely  2 | | | | | | | | Occasionally  3 | | | | | | | | | | | | Often  4 | | | | | | | | | | Very  Often  5 | | | | | | | | | | |  |
|  | | How often do you read the following types of newspapers__? | | | | | 1=never  2=rarely  3=occasionally  4=often  5=very often | | | | | | | | | | | | | | | | | | | | | | | | | | | | | | | | | | | | | | | | | | | | | | | | |  |
| 005d | | Sports | | | | | Never  1 | | | | | | | | Rarely  2 | | | | | | | | Occasionally  3 | | | | | | | | | | | | Often  4 | | | | | | | | | | Very  Often  5 | | | | | | | | | | |  |
| 005e | | News | | | | | Never  1 | | | | | | | | Rarely  2 | | | | | | | | Occasionally  3 | | | | | | | | | | | | Often  4 | | | | | | | | | | Very  Often  5 | | | | | | | | | | |  |
| 005f | | Religious | | | | | Never  1 | | | | | | | | Rarely  2 | | | | | | | | Occasionally  3 | | | | | | | | | | | | Often  4 | | | | | | | | | | Very  Often  5 | | | | | | | | | | |  |
| 005g | | Gossip | | | | | Never  1 | | | | | | | | Rarely  2 | | | | | | | | Occasionally  3 | | | | | | | | | | | | Often  4 | | | | | | | | | | Very  Often  5 | | | | | | | | | | |  |
| 005h | | Other | | | | | Never  1 | | | | | | | | Rarely  2 | | | | | | | | Occasionally  3 | | | | | | | | | | | | Often  4 | | | | | | | | | | Very  Often  5 | | | | | | | | | | |  |
| SECTION D: SOCIABILITY/ROLE MODELS | | | | | | | | | | | | | | | | | | | | | | | | | | | | | | | | | | | | | | | | | | | | | | | | | | | | | | | |  |
| 001 | | Do you belong to any music, dance or drama groups? | | | | | Yes 01 | | | | | | | | | | | | | | | | No 00  **SKIP TO 002** | | | | | | | | | | | | | | | | | | | | | | | | | | | | | | | | |  |
| 001a | | Which kinds (Circle all mentioned) | | | | | a. Traditional choirs  b. Traditional dance  c. Church choirs  d. Dramas  e. Other  f. DK  g. NA | | | | | | | | | | | | | | | | | | | | | | | | | | | | | | | | | | | | | | | | | | | | | | | | |  |
| 002 | | What types of activities or services brings temporary visitors to your villages  (Do not prompt)  (Circle all mentioned) | | | | | a. Construction e.g roads, wells, houses  b. Honey  c. Mining  d. Research  e. Selling merchandise  f. TANAPA  g. Wildlife Division  h. Anti-poaching of Hunting Companies  i. Truck drivers  j. Photo Tourism employees  k. Others (List) _______________  l. DK | | | | | | | | | | | | | | | | | | | | | | | | | | | | | | | | | | | | | | | | | | | | | | | | |  |
| 002a | | How often do you interact with temporary visitors to this village (Read choices) | | | | | Not at all  1 | | | | | | | | Rarely  2 | | | | | | | | Occasionally  3 | | | | | | | | | | | | Often  4 | | | | | | | | | | Very Often  5 | | | | | | | | | | |  |
| 003 | | What would you buy if you received the following amount of money? (Repeat question for each amount. Do not read options. Circle all mentioned.) | | | | **003a.TSH 10,000**  a. Clothing/shoes self  b. Clothing/shoes family  c. Food for family  d. Education  e. Livestock  f. Business  g. Other  **h. DK**   |  | | --- | |  | |  | | | | | | | | | | | **003b. TSH 50,000**  a. Clothing/shoes self  b. Clothing/shoes family  c. Food for family  d. Education  e. Livestock  f. Business  g. House  h. Phone  i. Furniture  j. Bicycle  k. Other  l. DK   |  | | --- | | | | | | | | | | | | | | | | | | | | **003c. TSH 100,000**  a. Clothing/shoes self  b. Clothing/shoes family  c. Food for family  d. Education  e. Business  f. Livestock  g. House  h. Phone  i. Bicycle  j. Farm  k. Furniture  l. Other  m. DK   |  | | --- | | | | | | | | | | | | | | | | | | | | | |  |
| 004 | | What kind of clothing would you most likely buy for yourself? | | | | 01- Traditional clothes e.g. kitenge/khanga  02- Ordinary clothes e.g. regular T-shirts, shirts, skirt, trousers  03- Fashionable clothes e.g. stylish/western clothes  77-NA | | | | | | | | | | | | | | | | | | | | | | | | | | | | | | | | | | | | | | | | |  | | | | | | | | |  |
| 005 | | Do you have a role model? | | | | Yes  01 | | | | | | | | | | | | | | | | No  00  **SKIP TO 006** | | | | | | | | | | | | | | | | | | | | | | | | | | | | | | | | | |  |
| 005a | | If yes, who? Listen to answer and probe for category CHOOSE ONE. PROBE IF UNKNOWN NAME MENTIONED AND PUT IN APPROPRIATE CATEGORY | | | | 01= Biological parent  02= Among relatives  03= Neighbor  04= Friend  05= Media figure/star  06= Elder brother/sister  07= Religious leader  08= Political leader  09= Teacher  10= Athletic  11= Wealth person  12= Traditional leader  99= Other | | | | | | | | | | | | | | | | | | | | | | | | | | | | | | | | | | | | | | | | | | | | | | | | | |  |
| 006 | | What would your parents do if you: (Read all 5 categories for each situation and circle answers to match answers below)  1 Extremely angry  2 Somewhat angry  3 Neither angry or  happy  4 Somewhat happy  5 Extremely happy | | | | a. Came home very late  1 2 3 4 5 | | | | | | | | | | | | | | | | | | | | | | | | | | | | | | | | | | | | | | | | | | | | | | | | | |  |
|  |  |  |  |  |  | b. Sleep out of your home  1 2 3 4 5 | | | | | | | | | | | | | | | | | | | | | | | | | | | | | | | | | | | | | | | | | | | | | | | | | |  |
|  |  |  |  |  |  | c. Find out you have a girl or boyfriend  1 2 3 4 5 | | | | | | | | | | | | | | | | | | | | | | | | | | | | | | | | | | | | | | | | | | | | | | | | | |  |
|  |  |  |  |  |  | d. Find out you are pregnant or got a girl pregnant?  1 2 3 4 5  . | | | | | | | | | | | | | | | | | | | | | | | | | | | | | | | | | | | | | | | | | | | | | | | | | |  |
|  |  |  |  |  |  | e. Find out you drank alcohol  1 2 3 4 5 | | | | | | | | | | | | | | | | | | | | | | | | | | | | | | | | | | | | | | | | | | | | | | | | | |  |
| 007 | | Who would you seek advice from about the following types of issues?   | 1=Father | | --- | | 2=Mother | | 3=Spouse/partner | | 4=  Brother | | 5=sister | | 6=     Brother/sister in-law | | 7=    Medical doctor | | 8=Friend | | 9=   Village elder | | 10=     Religious leader | | 11=Traditional leader | | 12=Teacher | | 99=. Other | | | | | a. Education | | | | | | | | | | | | | | | | | | | | | | | | | | | | | | | | | | | | | | | | | | | | | | | | | |  |
|  |  |  |  |  |  | b. Health issue | | | | | | | | | | | | | | | | | | | | | | | | | | | | | | | | | | | | | | | | | | | | | | | | | |  |
|  |  |  |  |  |  | c. Relationships | | | | | | | | | | | | | | | | | | | | | | | | | | | | | | | | | | | | | | | | | | | | | | | | | |  |
|  |  |  |  |  |  | d. Marriage | | | | | | | | | | | | | | | | | | | | | | | | | | | | | | | | | | | | | | | | | | | | | | | | | |  |
|  |  |  |  |  |  | e. Business | | | | | | | | | | | | | | | | | | | | | | | | | | | | | | | | | | | | | | | | | | | | | | | | | |  |
| **SECTION E:** GOALS AND ASPIRATIONS | | | | | | | | | | | | | | | | | | | | | | | | | | | | | | | | | | | | | | | | | | | | | | | | | | | | | | | |  |
| 001 | | How important to you is achieving the following goals during your life? | | | | | | | | | | | | | | | | | | | | | | | | | | | | | | | | | | | | | | | | | | | | | | | | | | | | | |  |
| 001a | | Own a farm | | | | Not at all important  1 | | | | | | | | Not very important  2 | | | | | | | Somewhat Important  3 | | | | | | | | | | | | | | Important  4 | | | | | | | | | | Extremely Important  5 | | | | | | | | | | |  |
| 001b | | Complete secondary school | | | | Not at all important  1 | | | | | | | | Not very important  2 | | | | | | | Somewhat Important  3 | | | | | | | | | | | | | | Important  4 | | | | | | | | | | Extremely Important  5 | | | | | | | | | | |  |
| 001c | | Open a business | | | | Not at all important  1 | | | | | | | | Not very important  2 | | | | | | | Somewhat Important  3 | | | | | | | | | | | | | | Important  4 | | | | | | | | | | Extremely Important  5 | | | | | | | | | | |  |
| 001d | | Get married | | | | Not at all important  1 | | | | | | | | Not very important  2 | | | | | | | Somewhat Important  3 | | | | | | | | | | | | | | Important  4 | | | | | | | | | | Extremely Important  5 | | | | | | | | | | |  |
| 001e | | Build a house | | | | Not at all important  1 | | | | | | | | Not very important  2 | | | | | | | Somewhat Important  3 | | | | | | | | | | | | | | Important  4 | | | | | | | | | | Extremely Important  5 | | | | | | | | | | |  |
| 001f | | Move to town | | | | Not at all important  1 | | | | | | | | Not very important  2 | | | | | | | Somewhat Important  3 | | | | | | | | | | | | | | Important  4 | | | | | | | | | | Extremely Important  5 | | | | | | | | | | |  |
| 001g | | Buy livestock | | | | Not at all important  1 | | | | | | | | Not very important  2 | | | | | | | Somewhat Important  3 | | | | | | | | | | | | | | Important  4 | | | | | | | | | | Extremely Important  5 | | | | | | | | | | |  |
| 001h | | Complete higher degree than secondary school | | | | Not at all important  1 | | | | | | | | Not very important  2 | | | | | | | Somewhat Important  3 | | | | | | | | | | | | | | Important  4 | | | | | | | | | | Extremely Important  5 | | | | | | | | | | |  |
| 001i | | Have children | | | | Not at all important  1 | | | | | | | | Not very important  2 | | | | | | | Somewhat Important  3 | | | | | | | | | | | | | | Important  4 | | | | | | | | | | Extremely Important  5 | | | | | | | | | | |  |
| 001j | | Become a teacher or another professional | | | | Not at all important  1 | | | | | | | | Not very important  2 | | | | | | | Somewhat Important  3 | | | | | | | | | | | | | | Important  4 | | | | | | | | | | Extremely Important  5 | | | | | | | | | | |  |
| 001k | | Become wealthy | | | | Not at all important  1 | | | | | | | | Not very important  2 | | | | | | | Somewhat Important  3 | | | | | | | | | | | | | | Important  4 | | | | | | | | | | Extremely Important  5 | | | | | | | | | | |  |
| 001l | | Live the “good life” in the city | | | | Not at all important  1 | | | | | | | | Not very important  2 | | | | | | | Somewhat Important  3 | | | | | | | | | | | | | | Important  4 | | | | | | | | | | Extremely Important  5 | | | | | | | | | | |  |
| 002 | | I am going to read you a number of characteristics that describe a person’s reputation. What traits would you most or least like to be known for? (Read each trait followed by the scale) | | | | | | | | | | | | | | | | | | | | | | | | | | | | | | | | | | | | | | | | | | | | | | | | | | | |  | |  |
| 002a | | Most athletic | | | | Not like at all  1 | | | | | | | | Somewhat dislike  2 | | | | | | | Neither like nor dislike  3 | | | | | | | | | | | | | | Somewhat like  4 | | | | | | | | | | Like a great deal  5 | | | | | | | | | | |  |
| 002b | | Most brave | | | | Not like at all  1 | | | | | | | | Somewhat dislike  2 | | | | | | | Neither like nor dislike  3 | | | | | | | | | | | | | | Somewhat like  4 | | | | | | | | | | Like a great deal  5 | | | | | | | | | | |  |
| 002c | | Prettiest/handsome | | | | Not like at all  1 | | | | | | | | Somewhat dislike  2 | | | | | | | Neither like nor dislike  3 | | | | | | | | | | | | | | Somewhat like  4 | | | | | | | | | | Like a great deal  5 | | | | | | | | | | |  |
| 002d | | Wealthiest | | | | Not like at all  1 | | | | | | | | Somewhat dislike  2 | | | | | | | Neither like nor dislike  3 | | | | | | | | | | | | | | Somewhat like  4 | | | | | | | | | | Like a great deal  5 | | | | | | | | | | |  |
| 002e | | Best dressed | | | | Not like at all  1 | | | | | | | | Somewhat dislike  2 | | | | | | | Neither like nor dislike  3 | | | | | | | | | | | | | | Somewhat like  4 | | | | | | | | | | Like a great deal  5 | | | | | | | | | | |  |
| 002f | | Most powerful | | | | Not like at all  1 | | | | | | | | Somewhat dislike  2 | | | | | | | Neither like nor dislike  3 | | | | | | | | | | | | | | Somewhat like  4 | | | | | | | | | | Like a great deal  5 | | | | | | | | | | |  |
| 002g | | Sexiest | | | | Not like at all  1 | | | | | | | | Somewhat dislike  2 | | | | | | | Neither like nor dislike  3 | | | | | | | | | | | | | | Somewhat like  4 | | | | | | | | | | Like a great deal  5 | | | | | | | | | | |  |
| 002h | | Most artistic | | | | Not like at all  1 | | | | | | | | Somewhat dislike  2 | | | | | | | Neither like nor dislike  3 | | | | | | | | | | | | | | Somewhat like  4 | | | | | | | | | | Like a great deal  5 | | | | | | | | | | |  |
| 002i | | Best in farming | | | | Not like at all  1 | | | | | | | | Somewhat dislike  2 | | | | | | | Neither like nor dislike  3 | | | | | | | | | | | | | | Somewhat like  4 | | | | | | | | | | Like a great deal  5 | | | | | | | | | | |  |
| 002j | | Most trustworthy | | | | Not like at all  1 | | | | | | | | Somewhat dislike  2 | | | | | | | Neither like nor dislike  3 | | | | | | | | | | | | | | Somewhat like  4 | | | | | | | | | | Like a great deal  5 | | | | | | | | | | |  |
| 002k | | Best friend to others | | | | Not like at all  1 | | | | | | | | Somewhat dislike  2 | | | | | | | Neither like nor dislike  3 | | | | | | | | | | | | | | Somewhat like  4 | | | | | | | | | | Like a great deal  5 | | | | | | | | | | |  |
| 002l | | Most intelligent | | | | Not like at all  1 | | | | | | | | Somewhat dislike  2 | | | | | | | Neither like nor dislike  3 | | | | | | | | | | | | | | Somewhat like  4 | | | | | | | | | | Like a great deal  5 | | | | | | | | | | |  |
| 002m | | Hardest worker | | | | Not like at all  1 | | | | | | | | Somewhat dislike  2 | | | | | | | Neither like nor dislike  3 | | | | | | | | | | | | | | Somewhat like  4 | | | | | | | | | | Like a great deal  5 | | | | | | | | | | |  |
| 002n | | Most fun | | | | Not like at all  1 | | | | | | | | Somewhat dislike  2 | | | | | | | Neither like nor dislike  3 | | | | | | | | | | | | | | Somewhat like  4 | | | | | | | | | | Like a great deal  5 | | | | | | | | | | |  |
| 002o | | Most traditional | | | | Not like at all  1 | | | | | | | | Somewhat dislike  2 | | | | | | | Neither like nor dislike  3 | | | | | | | | | | | | | | Somewhat like  4 | | | | | | | | | | Like a great deal  5 | | | | | | | | | | |  |
| 002p | | Most helpful to others | | | | Not like at all  1 | | | | | | | | Somewhat dislike  2 | | | | | | | Neither like nor dislike  3 | | | | | | | | | | | | | | Somewhat like  4 | | | | | | | | | | Like a great deal  5 | | | | | | | | | | |  |
| 002q | | Most religious | | | | Not like at all  1 | | | | | | | | Somewhat dislike  2 | | | | | | | Neither like nor dislike  3 | | | | | | | | | | | | | | Somewhat like  4 | | | | | | | | | | Like a great deal  5 | | | | | | | | | | |  |
| 002r | | Best dancer | | | | Not like at all  1 | | | | | | | | Somewhat dislike  2 | | | | | | | Neither like nor dislike  3 | | | | | | | | | | | | | | Somewhat like  4 | | | | | | | | | | Like a great deal  5 | | | | | | | | | | |  |
| 002s | | Best hunter | | | | Not like at all  1 | | | | | | | | Somewhat dislike  2 | | | | | | | Neither like nor dislike  3 | | | | | | | | | | | | | | Somewhat like  4 | | | | | | | | | | Like a great deal  5 | | | | | | | | | | |  |
| 002t | | Best student | | | | Not like at all  1 | | | | | | | | Somewhat dislike  2 | | | | | | | Neither like nor dislike  3 | | | | | | | | | | | | | | Somewhat like  4 | | | | | | | | | | Like a great deal  5 | | | | | | | | | | |  |
| 002u | | Best at doing business | | | | Not like at all  1 | | | | | | | | Somewhat dislike  2 | | | | | | | Neither like nor dislike  3 | | | | | | | | | | | | | | Somewhat like  4 | | | | | | | | | | Like a great deal  5 | | | | | | | | | | |  |
| 002v | | Best leader | | | | Not like at all  1 | | | | | | | | Somewhat dislike  2 | | | | | | | Neither like nor dislike  3 | | | | | | | | | | | | | | Somewhat like  4 | | | | | | | | | | Like a great deal  5 | | | | | | | | | | |  |
| 002w | | Best livestock keeper | | | | Not like at all  1 | | | | | | | | Somewhat dislike  2 | | | | | | | Neither like nor dislike  3 | | | | | | | | | | | | | | Somewhat like  4 | | | | | | | | | | Like a great deal  5 | | | | | | | | | | |  |
| 002x | | Most creative | | | | Not like at all  1 | | | | | | | | Somewhat dislike  2 | | | | | | | Neither like nor dislike  3 | | | | | | | | | | | | | | Somewhat like  4 | | | | | | | | | | Like a great deal  5 | | | | | | | | | | |  |
| 002y | | Most polite | | | | Not like at all  1 | | | | | | | | Somewhat dislike  2 | | | | | | | Neither like nor dislike  3 | | | | | | | | | | | | | | Somewhat like  4 | | | | | | | | | | Like a great deal  5 | | | | | | | | | | |  |
| 002z | | Most ambitious | | | | Not like at all  1 | | | | | | | | Somewhat dislike  2 | | | | | | | Neither like nor dislike  3 | | | | | | | | | | | | | | Somewhat like  4 | | | | | | | | | | Like a great deal  5 | | | | | | | | | | |  |
| 002z1 | | Most responsible | | | | Not like at all  1 | | | | | | | | Somewhat dislike  2 | | | | | | | Neither like nor dislike  3 | | | | | | | | | | | | | | Somewhat like  4 | | | | | | | | | | Like a great deal  5 | | | | | | | | | | |  |
| 002z2 | | Most talkative | | | | Not like at all  1 | | | | | | | | Somewhat dislike  2 | | | | | | | Neither like nor dislike  3 | | | | | | | | | | | | | | Somewhat like  4 | | | | | | | | | | Like a great deal  5 | | | | | | | | | | |  |
| I would like to know what you believe about different things. Tell me if you strongly disagree, disagree, neither agree or disagree, agree or strongly agree with the following statements: | | | | | | | | | | | | | | | | | | | | | | | | | | | | | | | | | | | | | | | | | | | | | | | | | | | | | | | |  |
| 003 | | I have everything I need to achieve my goals. | Strongly  Disagree  1 | | | | | | | | Disagree  2 | | | | | | Neither Agree or Disagree  3 | | | | | | | | | | | | Agree  4 | | | | | | | | | | | | Strongly agree  5 | | | | | | | | | | |  | | | |  |
| 004 | | There are few prospects for me in this village | Strongly  Disagree  1 | | | | | | | | Disagree  2 | | | | | | Neither Agree or Disagree  3 | | | | | | | | | | | | Agree  4 | | | | | | | | | | | | Strongly agree  5 | | | | | | | | | | |  | | | |  |
| 005 | | People are usually faithful to their partners | Strongly  Disagree  1 | | | | | | | | Disagree  2 | | | | | | Neither Agree or Disagree  3 | | | | | | | | | | | | Agree  4 | | | | | | | | | | | | Strongly agree  5 | | | | | | | | | | |  | | | |  |
| 006 | | If I pray and remain faithful to God I will succeed | Strongly  Disagree  1 | | | | | | | | Disagree  2 | | | | | | Neither Agree or Disagree  3 | | | | | | | | | | | | Agree  4 | | | | | | | | | | | | Strongly agree  5 | | | | | | | | | | |  | | | |  |
| 007. | | I trust my partner to always be faithful | Strongly  Disagree  1 | | | | | | | | Disagree  2 | | | | | | Neither Agree or Disagree  3 | | | | | | | | | | | | Agree  4 | | | | | | | | | | | | Strongly agree  5 | | | | | | | | | | |  | | | |  |
| 008 | | People who drink alcohol are often in trouble | Strongly  Disagree  1 | | | | | | | | Disagree  2 | | | | | | Neither Agree or Disagree  3 | | | | | | | | | | | | Agree  4 | | | | | | | | | | | | Strongly agree  5 | | | | | | | | | | |  | | | |  |
| 009 | | Most married people have other long-term relationships at the same time | Strongly  Disagree  1 | | | | | | | | Disagree  2 | | | | | | Neither Agree or Disagree  3 | | | | | | | | | | | | Agree  4 | | | | | | | | | | | | Strongly agree  5 | | | | | | | | | | |  | | | |  |
| 010 | | Government support is extremely important to my success | Strongly  Disagree  1 | | | | | | | | Disagree  2 | | | | | | Neither Agree or Disagree  3 | | | | | | | | | | | | Agree  4 | | | | | | | | | | | | Strongly agree  5 | | | | | | | | | | |  | | | |  |
| 011 | | It is better to work smart than to work hard to become wealthy | Strongly  Disagree  1 | | | | | | | | Disagree  2 | | | | | | Neither Agree or Disagree  3 | | | | | | | | | | | | Agree  4 | | | | | | | | | | | | Strongly agree  5 | | | | | | | | | | |  | | | |  |
| 012 | | Some people must participate in dishonest activities just to get by | Strongly  Disagree  1 | | | | | | | | Disagree  2 | | | | | | Neither Agree or Disagree  3 | | | | | | | | | | | | Agree  4 | | | | | | | | | | | | Strongly agree  5 | | | | | | | | | | |  | | | |  |
| 013 | | If everyone worked together our lives will improve. | Strongly  Disagree  1 | | | | | | | | Disagree  2 | | | | | | Neither Agree or Disagree  3 | | | | | | | | | | | | Agree  4 | | | | | | | | | | | | Strongly agree  5 | | | | | | | | | | |  | | | |  |
| 014 | | Education and study are the best way to succeed in life | Strongly  Disagree  1 | | | | | | | | Disagree  2 | | | | | | Neither Agree or Disagree  3 | | | | | | | | | | | | Agree  4 | | | | | | | | | | | | Strongly agree  5 | | | | | | | | | | |  | | | |  |
| 015 | | My parents and family are most important to my success | Strongly  Disagree  1 | | | | | | | | Disagree  2 | | | | | | Neither Agree or Disagree  3 | | | | | | | | | | | | Agree  4 | | | | | | | | | | | | Strongly agree  5 | | | | | | | | | | |  | | | |  |
| 016 | | To fulfill my life goals, I will need to live in a city | Strongly  Disagree  1 | | | | | | | | Disagree  2 | | | | | | Neither Agree or Disagree  3 | | | | | | | | | | | | Agree  4 | | | | | | | | | | | | Strongly agree  5 | | | | | | | | | | |  | | | |  |
| 017 | | My success depends mostly on my own efforts | Strongly  Disagree  1 | | | | | | | | Disagree  2 | | | | | | Neither Agree or Disagree  3 | | | | | | | | | | | | Agree  4 | | | | | | | | | | | | Strongly agree  5 | | | | | | | | | | |  | | | |  |
| **SECTION F:** SEXUAL PRACTICES - ENTIRELY NVRC (LINDBERG CARDS) - EXPECT 9G, 9L, 9R, 9T AND 9V | | | | | | | | | | | | | | | | | | | | | | | | | | | | | | | | | | | | | | | | | | | | | | | | | | | | | | | |  |
| 001 | | Have you ever had sexual intercourse? | | | | | Yes  01 | | | | | | | | | | | | | | | | | | | | | | | | | No 00  Not answered 66  **SKIP TO 007** | | | | | | | | | | | | | | | | | | | | | | | |  |
| 002 | | What age were you when you first had sexual intercourse? | | | | | __________________ | | | | | | | | | | | | | | | | | | | | | | | | | | | | | | | | | | | | | | | | | | | | | | | | |  |
| 003 | | How many sexual partners have you had in your whole life? | | | | | ___________________ | | | | | | | | | | | | | | | | | | | | | | | | | | | | | | | | | | | | | | | | | | | | | | | | |  |
| 004 | | How many sexual partners have you had in the past 3 months? | | | | | ___________________ | | | | | | | | | | | | | | | | | | | | | | | | | | | | | | | | | | | | | | | | | | | | | | | | |  |
| 005 | | The last time you had sexual intercourse, was a condom used? | | | | | 01 Yes  00 No  02 I have never used a condom 🡺 **SKIP TO 007** | | | | | | | | | | | | | | | | | | | | | | | | | | | | | | | | | | | | | | | | | | | | | | | | |  |
| 006 | | Was a condom used every time you had sexual intercourse in the past twelve months? | | | | | 01 Yes  00 No | | | | | | | | | | | | | | | | | | | | | | | | | | | | | | | | | | | | | | | | | | | | | | | | |  |
| 007 | | Do you know a place where you could get a condom  ? | | | | | 01 Yes  00 No | | | | | | | | | | | | | | | | | | | | | | | | | | | | | | | | | | | | | | | | | | | | | | | | |  |
| 008 | | If you wanted to, could you yourself get a condom? | | | | | 01 Yes  00 No_  88 Don’t know | | | | | | | | | | | | | | | | | | | | | | | | | | | | | | | | | | | | | | | | | | | | | | | | |  |
| 009**. Are you interviewing a female/girl?**  **00 No 🡺 SKIP TO 009L**  **01 Yes** | | | | | | | | | | | | | | | | | | | | | | | | | | | | | | | | | | | | | | | | | | | | | | | | | | | | | | | |  |
| 009a | | How many pregnancies have you had so far? | | | | | ___________________ IF “0” SKIP TO 009F | | | | | | | | | | | | | | | | | | | | | | | | | | | | | | | | | | | | | | | | | | | | | | | | |  |
| 009b | | How old were you at first pregnancy? | | | | | _______________ | | | | | | | | | | | | | | | | | | | | | | | | | | | | | | | | | | | | | | | | | | | | | | | | |  |
| 009c | | How many births have you had so far? | | | | | _______________ | | | | | | | | | | | | | | | | | | | | | | | | | | | | | | | | | | | | | | | | | | | | | | | | |  |
| 009d | | With how many different men have you had pregnancies with? | | | | | _______________ | | | | | | | | | | | | | | | | | | | | | | | | | | | | | | | | | | | | | | | | | | | | | | | | |  |
| 009e | | How many kids are still living? | | | | | _______________ IF ANS IS “0” SKIP TO 009g | | | | | | | | | | | | | | | | | | | | | | | | | | | | | | | | | | | | | | | | | | | | | | | | |  |
| 009f | | How many kids are living with you now? | | | | | _______________ | | | | | | | | | | | | | | | | | | | | | | | | | | | | | | | | | | | | | | | | | | | | | | | | |  |
| 009g | | What is your marital status? | | | | | 01.married or living together  02. divorced or separated  03. widowed   1. never married 🡺SKIP TO NEXT SECTION   99. other | | | | | | | | | | | | | | | | | | | | | | | | | | | | | | | | | | | | | | | | | | | | | | | | |  |
| 009h | | How old were you when you married? | | | | | _________________ | | | | | | | | | | | | | | | | | | | | | | | | | | | | | | | | | | | | | | | | | | | | | | | | |  |
| 009i | | Is your husband married polygynously | | | | | 00 No **SKIP TO 009K**  01 Yes | | | | | | | | | | | | | | | | | | | | | | | | | | | | | | | | | | | | | | | | | | | | | | | | |  |
| 009j | | If so how many co-wives? | | | | | ____________ | | | | | | | | | | | | | | | | | | | | | | | | | | | | | | | | | | | | | | | | | | | | | | | | |  |
| 009k | | If so what wife number are you? | | | | | ____________ | | | | | | | | | | | | | | | | | | | | | | | | | | | | | | | | | | | | | | | | | | | | | | | | |  |
| 009l | | What role did you have in choice of first husband? | | | | | 1= none  2= parents (wazazi-wazee) gave strong advice  3= Parent’s approval  4= parents consultation  5= free choice  6= Caused by pregnancy | | | | | | | | | | | | | | | | | | | | | | | | | | | | | | | | | | | | | | | | | | | | | | | | |  |
| **009. For male** | | | | | | | | | | | | | | | | | | | | | | | | | | | | | | | | | | | | | | | | | | | | | | | | | | | | | | | |  |
| 009m | | How many children have you produced? | | | | | ________________ IF ANS =0, SKIP TO 009R | | | | | | | | | | | | | | | | | | | | | | | | | | | | | | | | | | | | | | | | | | | | | | | | |  |
| 009n | | How old were you at first child's birth? | | | | | ________________ | | | | | | | | | | | | | | | | | | | | | | | | | | | | | | | | | | | | | | | | | | | | | | | | |  |
| 009o | | How many are still living | | | | | _______________ | | | | | | | | | | | | | | | | | | | | | | | | | | | | | | | | | | | | | | | | | | | | | | | | |  |
| 009p | | How many different mothers did you have with? | | | | | _______________ | | | | | | | | | | | | | | | | | | | | | | | | | | | | | | | | | | | | | | | | | | | | | | | | |  |
| 009q | | How many are you living with you now? | | | | | ______________ | | | | | | | | | | | | | | | | | | | | | | | | | | | | | | | | | | | | | | | | | | | | | | | | |  |
| 009r | | What is your marital status? | | | | | 01. married or living together  02. divorced or separated  03. widowed  04. never married 🡺 SKIP TO NEXT SECTION  99. other | | | | | | | | | | | | | | | | | | | | | | | | | | | | | | | | | | | | | | | | | | | | | | | | |  |
| 009s | | How old were you when you married? | | | | | ___________ | | | | | | | | | | | | | | | | | | | | | | | | | | | | | | | | | | | | | | |  | | | | | | | | | |  |
| 009t | | Are you married polygynously | | | | | 00 No 🡺 SKIP TO 009V  01 Yes | | | | | | | | | | | | | | | | | | | | | | | | | | | | | | | | | | | | | | |  | | | | | | | | | |  |
| 009u | | If so how many wives do you have? | | | | | _____________ | | | | | | | | | | | | | | | | | | | | | | | | | | | | | | | | | | | | | | |  | | | | | | | | | |  |
| 009v | | What role did you have in choice of first wife? | | | | | 1=none  2=parents (wazazi-wazee) gave strong advice  3= parents approval  4=parents consultation  5=free choice  6= Caused by pregnancy | | | | | | | | | | | | | | | | | | | | | | | | | | | | | | | | | | | | | | | | | | | | | | | | |  |
| **SECTION G: HIV/AIDS -** ENTIRELY NVRC (LINDBERG CARDS) EXCEPT QUESTIONS 002, 004, 022 AND 023 | | | | | | | | | | | | | | | | | | | | | | | | | | | | | | | | | | | | | | | | | | | | | | | | | | | | | | | |  |
| 001 | | Have you ever heard of HIV or AIDS? | Yes  01 | | | | | | | | | | | | | | | | | | | | | | | No  00  SKIP TO 003 | | | | | | | | | | | | | | | | | | | | | | | | | |  | | | |  |
| 002 | | From what source did you learn about HIV/AIDS?  CHECK ALL THAT APPLY | | | a. Primary school  b. Secondary school  c. Posters  d. Television  e. Radio  f. Phone text | | | | | | | | | | | Y/N  Y/N  Y/N  Y/N  Y/N  Y/N | | | | | | g. Friends  h. Parents/relatives  i. Magazines  j. Performance  k. Other (specify)  l. DK  m. NA | | | | | | | | | | | | | | | | | | | | | | | | Y/N  Y/N  Y/N  Y/N  Y/N  Y/N  Y/N | | | | | | |  | | | |
| 003 | | Is there anything a person can do to avoid getting AIDS or the virus that causes AIDS? | Yes  01 | | | | | | | | | | | | | | | | | | | | | | No  00  SKIP TO 005 | | | | | | | | | | | | | 88 Don’t know  SKIP TO 005 | | | | | | | | | | | | | |  | | | |  |
| 004 | | What can a person do? ALLOW MORE THAN ONE RESPONSE. PROMPT: “Anything else?” BUT DO NOT READ | - 1. Abstain from sex 1 Yes 0 No   2. Use condoms always whenever having sex with casual partner 1 Yes 0 No   3. Limit sex to one partner who is not infected /stay faithful to one partner who is not infected 1 Yes 0 No   4. Avoid sex with prostitutes 1 Yes 0 No   5. Avoid sex with persons who have many partners 1 Yes 0 No   6. Avoid sex with persons who inject drugs intravenously 1 Yes 0 No   7. Avoid blood transfusions 1 Yes 0 No   8. Avoid unsafe injections 1 Yes 0 No   9. Avoid sharing sharps instruments like razors/blades 1 Yes 0 No   10. Seek protection from traditional practitioners 1 Yes 0 No   11. Other Yes 0 No | | | | | | | | | | | | | | | | | | | | | | | | | | | | | | | | | | | | | | | | | | | | | | | | | | | | |  |
| 005 | | Can people reduce their chances of getting the AIDS virus by having just one sex partner who has no other partners? | 01 Yes | | | | | | | | | | | | | | | | | | | | | | | | 00 No | | | | | | | | | | | | 88 Don’t know | | | | | | | | | | | | |  | | | |  |
| 006 | | Can people get the AIDS virus from mosquito bites? | 01 Yes | | | | | | | | | | | | | | | | | | | | | | | | 00 No | | | | | | | | | | | | 88 Don’t know | | | | | | | | | | | | |  | | | |  |
| 007 | | Can people reduce their chances of getting the AIDS virus by using a condom every time they have sex? | 01 Yes | | | | | | | | | | | | | | | | | | | | | | | | 00 No | | | | | | | | | | | | 88 Don’t know | | | | | | | | | | | | |  | | | |  |
| 008 | | Can people get the AIDS virus by sharing food with a person who has AIDS? | 01 Yes | | | | | | | | | | | | | | | | | | | | | | | | 00 No | | | | | | | | | | | | 88 Don’t know | | | | | | | | | | | | |  | | | |  |
| 009 | | Can ` HIV/AIDS can be avoided with vaccination? | 01 Yes | | | | | | | | | | | | | | | | | | | | | | | | 00 No | | | | | | | | | | | | 88 Don’t know | | | | | | | | | | | | |  | | | |  |
| 010 | | Is it possible for a healthy-looking person to have the AIDS virus? | 01 Yes | | | | | | | | | | | | | | | | | | | | | | | | 00 No | | | | | | | | | | | | 88 Don’t know | | | | | | | | | | | | |  | | | |  |
| 011 | | Is it possible for a child to contact hiv/aids from his/her mother via pregnancy? | 01 Yes | | | | | | | | | | | | | | | | | | | | | | | | 00 No | | | | | | | | | | | | 88 Don’t know | | | | | | | | | | | | |  | | | |  |
| 012 | | Is it possible for a child to contact hiv/aids from his/her mother at parturition? | 01 Yes | | | | | | | | | | | | | | | | | | | | | | | | 00 No | | | | | | | | | | | | 88 Don’t know | | | | | | | | | | | | |  | | | |  |
| 013 | | Is it possible for a child to contact hiv/aids from his/her mother via breastfeeding? | 01 Yes | | | | | | | | | | | | | | | | | | | | | | | | 00 No | | | | | | | | | | | | 88 Don’t know | | | | | | | | | | | | |  | | | |  |
| 014 | | Is HIV/AIDS caused by witchcraft? | 01 Yes | | | | | | | | | | | | | | | | | | | | | | | | 00 No | | | | | | | | | | | | 88 Don’t know | | | | | | | | | | | | |  | | | |  |
| 015 | | Is there treatment for AIDS? | 01 Yes | | | | | | | | | | | | | | | | | | | | | | | | 00 No | | | | | | | | | | | | 88 Don’t know | | | | | | | | | | | | |  | | | |  |
| 016 | | Do you personally know anyone who has died of AIDS? | | | Yes  01 | | | | | | | | | | | | | | | | | | No  00 | | | | | | | | | | | | | | 88 Don’t know | | | | | | | | | | | | | | | |  | |  | |
| 017 | | Do you personally know anyone currently ill with HIV/AIDS | | | Yes  01 | | | | | | | | | | | | | | | | | | No  00 | | | | | | | | | | | | | | 88 Don’t know | | | | | | | | | | | | | | | |  | |  | |
| 018 | Are you currently married, or do you have a sexual partner? | | | Yes  01 | | | | | | | | | | | | | | | No  00 🡺SKIP TO 019 | | | | | | | | | | | | | | | | | | | | | | | | | | | | | | |  | | | | |  | |
| 019 | If has a partner; Have you ever talked about ways to prevent getting the virus that causes AIDS with your husband/wife/partner? | | | Yes  01 | | | | | | | | | | | | | | | No  00 | | | | | | | | | | | | | | | | | | | | | | | | | | | | | | |  | | | | |  | |
| 020 | If a member of your family got infected with the virus that causes AIDS, would you be willing to care for her or him in your own household? | | | Yes  01 | | | | | | | | | | | | | | | No  00 | | | | | | | | | | | | | | | | | | | | | | | | | | | | | | | 88 Don’t know/not sure/it depends | | | | |  | |
| 021 | Would you buy fresh vegetables/food from a seller who has the AIDS virus? | | | Yes  01 | | | | | | | | | | | | | | | No  00 | | | | | | | | | | | | | | | | | | | | | | | | | | | | | | | 88 Don’t know/not sure/it depends | | | | |  | |
| 022 | Tell me if you strongly agree, agree, disagree, or strongly disagree with the following statements: A teacher with the AIDS virus who is not sick should be allowed to continue teaching. | | | 1. I strongly agree 2. I agree 3. I Disagree 4. I strongly disagree   88 Don’t know  66 Not answered | | | | | | | | | | | | | | | | | | | | | | | | | | | | | | | | | | | | | | | | | | | | | |  | | | | |  | |
| 023 | Tell me if you strongly agree, agree, disagree, or strongly disagree with the following statements: If a family member had the AIDS virus, that should be kept secret. | | | 1. I strongly agree 2. I agree 3. I Disagree 4. I strongly disagree   88 Don’t know  66 Not answered | | | | | | | | | | | | | | | | | | | | | | | | | | | | | | | | | | | | | | | | | | | | | |  | | | | |  | |
| 024 | Have you been tested for HIV/AIDS? | | | Yes  01 | | | | | | | | | | | | | | | No  00 – Skip to 024d | | | | | | | | | | | | | | | | | | | | | | | | | | | | | | |  | | | | |  | |
| 024a | IF TESTED: Did you receive the results? | | | Yes  01 | | | | | | | | | | | | | | | No  00 | | | | | | | | | | | | | | | | | | | | | | | | | | | | | | |  | | | | |  | |
| 024b | If yes; How many times? | | | 1  2  3  4 or more | | | | | | | | | | | | | | | 1  2  3  4 | | | | | | | | | | | | | | | | | | | | | | | | | | | | | | |  | | | | |  | |
| 024c | When was the last time you were tested? | | |  | | | | | | | | | | | | | | |  | | | | | | | | | | | | | | | | | | | | | | | | | | | | | | |  | | | | |  | |
| 024c1 | If yes; was it one past month the last time you tested? | | | Yes  01  00 – Skip to 024d | | | | | | | | | | | | | | | No | | | | | | | | | | | | | | | | | | | | | | | | | | | | | | |  | | | | |  | |
| 024c2 | If yes; was it one past year the last time you tested? | | | Yes  01  00 – Skip to 024d | | | | | | | | | | | | | | | No  00 | | | | | | | | | | | | | | | | | | | | | | | | | | | | | | |  | | | | |  | |
| 024c3 | If yes; was it more than one year the last time you tested? | | | Yes  01  00 – Skip to 024d | | | | | | | | | | | | | | | No  00 – Skip to 024d | | | | | | | | | | | | | | | | | | | | | | | | | | | | | | |  | | | | |  | |
| 024c4 | If yes; Don't you know the last time you tested? | | | Yes  01  00 – Skip to 024d | | | | | | | | | | | | | | | No  00 | | | | | | | | | | | | | | | | | | | | | | | | | | | | | | |  | | | | |  | |
| 024d | Has your partner been tested | | | Yes  No  DK  NA | | | | | | | | | | | | | | | 01  00 SKIP TO 025  88 SKIP TO 025  77 SKIP TO 025 | | | | | | | | | | | | | | | | | | | | | | | | | | | | | | |  | | | | |  | |
| 024e | When was the last time he/she was tested | | |  | | | | | | | | | | | | | | |  | | | | | | | | | | | | | | | | | | | | | | | | | | | | | | |  | | | | |  | |
| 024e1 | If yes; was it one past month the last time he/she tested? | | | Yes  01  Skip to 025 | | | | | | | | | | | | | | | No | | | | | | | | | | | | | | | | | | | | | | | | | | | | | | |  | | | | |  | |
| 024e2 | If yes; was it one past year the last time he/she tested? | | | Yes  01  Skip to 025 | | | | | | | | | | | | | | | No  00 | | | | | | | | | | | | | | | | | | | | | | | | | | | | | | |  | | | | |  | |
| 024e3 | If yes; was it more than one year the last time he/she tested? | | | Yes  01  Skip to 025 | | | | | | | | | | | | | | | No  00 – Skip to 024d | | | | | | | | | | | | | | | | | | | | | | | | | | | | | | |  | | | | |  | |
| 024e4 | If yes; Don't you know the last time he/she tested? | | | Yes  01  Skip to 025 | | | | | | | | | | | | | | | No  00 | | | | | | | | | | | | | | | | | | | | | | | | | | | | | | |  | | | | |  | |

| 025 Translator used (1=not at all, 2=sometimes, 3=all the time) | | |
| --- | --- | --- |
| 026 Language used in interview | Use national language list |  |
| 027 Result codes | | 01 Completed  02 Not at home  03 Refused  04 Partly completed |
